# Surpassing the 10% Efficiency Threshold in Perovskite-Inspired Indoor Photovoltaics

**DOI:** 10.1021/acsenergylett.5c01472

**Published:** 2025-06-25

**Authors:** Noora Lamminen, Jussi Lahtinen, Mokurala Krishnaiah, Joshua Karlsson, Milan Saju, G. Krishnamurthy Grandhi, Paola Vivo

**Affiliations:** Hybrid Solar Cells, Faculty of Engineering and Natural Sciences, 7840 Tampere University, P.O. Box 541, Tampere, FI-33014, Finland

## Abstract

Perovskite-inspired materials (PIMs) are promising candidates for low-toxicity indoor photovoltaics (IPVs), but their power conversion efficiencies (PCEs) have been so far largely limited by poor thin-film morphology and suboptimal device architectures. Here, we report a PCE exceeding 10% under 1000 lux indoor lighting by integrating device and film engineering strategies, including a dual-purpose interfacial modifier atop a hybrid antimony–bismuth halide PIM, advancing the development of efficient pnictogen-based IPVs for Internet of Things (IoT) applications.

## Graphical abstract

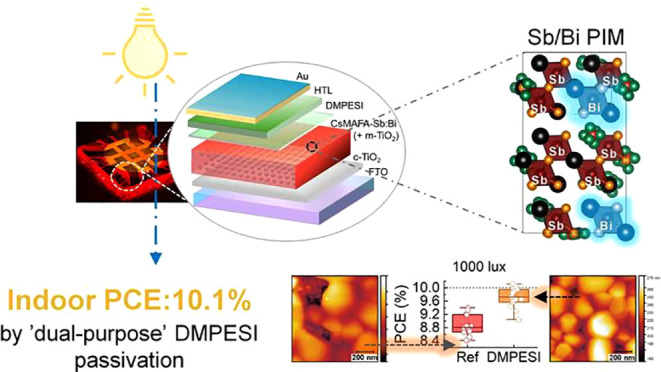

The growing demand for self-powered Internet of Things (IoT) devices has renewed interest in indoor photovoltaics (IPVs), which harvest ambient light as a sustainable energy source, reducing reliance on environmentally problematic batteries.^1^

Among emerging IPV technologies, lead halide perovskites (LHPs) have recently surpassed 44% power conversion efficiency (PCE) at 1000 lux.^2^ In contrast, lead-free perovskite-inspired materials (PIMs), especially those based on bismuth (Bi) and antimony (Sb), offer low toxicity,^3,4^ cost-effectiveness, and wide bandgaps, with theoretical efficiencies exceeding 50%, making them particularly suitable for indoor applications in close-contact environments.^1^

Experimentally, however, PIM-based IPV devices have shown modest PCEs around 5%, with a record of 7.6% for Cs_2_AgBi_2_I_9_ under standard indoor lighting conditions (1000 lux, ∼300 μW cm^–2^).^5^ These limitations stem from low short-circuit current density (*J*_SC_) and open-circuit voltage (*V*_OC_), due to suboptimal device configurations, high defect densities, and poor film morphologies.^6^ While mesoscopic architectures and hole transport layer (HTL) engineering have been explored,^5,7,8^ they have yet to yield major efficiency breakthroughs in Sb- and Bi-PIMs.

Achieving a 10% indoor PCE represents a critical milestone for establishing PIMs as viable, sustainable IPV materials, and it provides strong justification for their continued development. At this efficiency level, devices with active areas of 0.1–1.0 cm^2^ can generate ∼ 3–30 μW under 1000 lux—sufficient to intermittently power low-duty-cycle IoT devices such as Bluetooth Low Energy (BLE) beacons, sensors, and e-ink displays when paired with energy storage.^9^ Reaching this benchmark requires minimizing losses from morphological nonuniformity and interfacial defects, while optimizing architectures for indoor lighting to enhance absorption, charge transport, and overall efficiency.

To address these performance limitations, we selected a triple A-site cation Sb–Bi mixed PIM, CsMAFA-Sb:Bi, which has recently demonstrated remarkable operational stability under various stress conditions.^10^ Operational stability is essential for practical deployment, and among reported PIMs, CsMAFA-Sb:Bi currently stands out as the most promising candidate. In this work, we combined a mesoscopic architecture with HTL screening and introduced a dual-purpose interfacial modifier between the CsMAFA-Sb:Bi PIM layer and the HTL. This modifier simultaneously passivates surface traps and improves interfacial morphology—enhancing IPV performance. Notably, such an interfacial strategy has not previously been applied to PIMs, particularly in the context of IPVs.

We initially selected poly­[(2,5-bis­(2-hexyldecyloxy)-phenylene)-*alt*-(5,6-difluoro 4,7-di­(thiophen-2-yl)­benzo­[*c*]-[1,2,5]-thiadiazole)] (PPDT2FBT) as the HTL due to its proven effectiveness in enhancing the IPV performance of Cs_2_AgBi_2_I_9_.^5^ Mesoscopic CsMAFA-Sb:Bi devices (Figure 1A) incorporating PPDT2FBT achieved an average PCE of 3.5% under 1-Sun, outperforming planar counterparts (2.6%) (Figure 1B). Adding a thin dimethylphenethylsulfonium iodide (DMPESI) layer atop the absorber boosted average PCE to 4.1%, with a peak of 4.4% (Figures 1B and S1). Following optimization of the mesoporous TiO_2_ (m-TiO_2_) concentration, a PCE of ∼7.5% was achieved under 1000 lux white light-emitting diode (WLED) lighting (Figures S2–S4).

**Figure 1:**
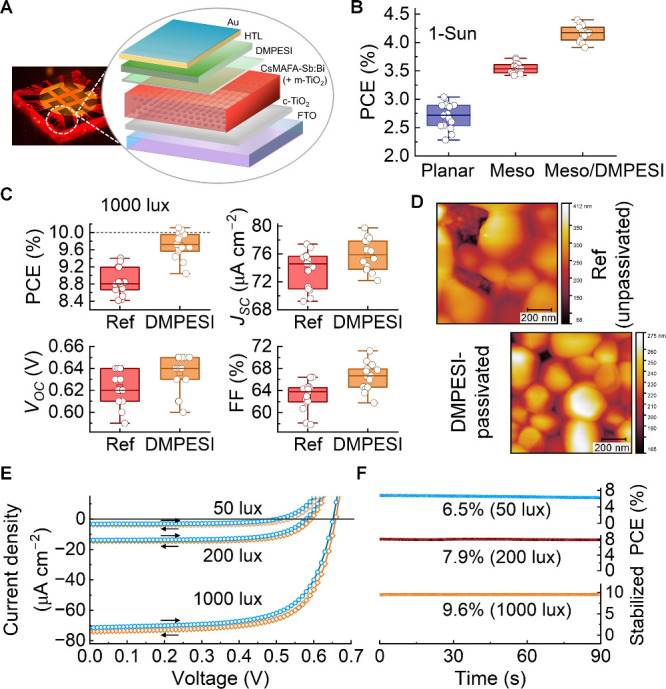
(A) Photograph of a substrate with eight photovoltaic devices under bottom white-light illumination, and a schematic of the mesoscopic device architecture incorporating a thin DMPESI layer atop the CsMAFA-Sb:Bi PIM layer. (B) PCE distributions of planar and mesoscopic devices, with and without a DMPESI layer, all employing PPDT2FBT as the HTL. (C) Distributions of PCE, *J*_SC_, *V*_OC_, and FF for unpassivated (“ref”) and DMPESI-passivated (“DMPESI”) devices (10 mm^2^ active area) using Spiro-OMeTAD as the HTL, under 1000 lux WLED illumination (6500 K). (D) Representative AFM topography images of CsMAFA-Sb:Bi films on TiO_2_ without and with DMPESI surface passivation. (E) Reverse and forward current density–voltage (*J–V*) curves (arrows indicate scan direction) of the champion DMPESI-passivated device (10 mm^2^ active area) under 1000, 200, and 50 lux WLED (6500 K) illumination. (F) Corresponding stabilized PCE curves obtained via maximum power point (MPP) tracking at each light intensity.

Among the HTLs tested, 2,2’,7,7’-tetrakis­(*N*,*N*-di-*p*-methoxyphenylamine)-9,9’-spirobifluorene (Spiro-OMeTAD) performed comparably to PPDT2FBT under 1-Sun (Figure S5), but surpassed it under 1000 lux, achieving 9.5% PCE (Figure S6). Further optimization of the absorber and m-TiO_2_ layers—yielding apparent thicknesses of ∼400 nm and ∼ 200 nm, respectively (Figure S7)—enabled indoor PCEs exceeding 10% (Supplementary Note 1, Figure S8). Previous reports on Sb/Bi PIMs, such as Cu_2_AgBiI_6_, Cs_2_AgBi_2_I_9_, and A_3_Sb_2_I_9_, have reached up to 7.6% PCE^1,5,6^ under 1000 lux WLED (standard ∼ 300 μW cm^–2^ irradiance), underscoring the significance of the >10% PCE demonstrated herein.

Introducing the DMPESI interlayer on the CsMAFA-Sb:Bi absorber significantly enhanced device performance, increasing the average PCE from 8.85% to 9.70% at 1000 lux (Figure 1C, Table S1), with similar improvements observed at both 50 and 200 lux (Figures S9 and S10).

X-ray photoelectron spectroscopy (XPS) confirmed chemical interactions between DMPESI and the CsMAFA-Sb:Bi surface, indicating effective surface passivation (Supplementary Note 2, Figures S11–S13, Table S2). Atomic force microscopy (AFM) revealed reduced surface roughness and waviness at the PIM/Spiro-OMeTAD interface (Table S3), along with improved film coverage after passivation (Figure 1D), corroborated by scanning electron microscopy (SEM) (Figure S14). These results suggest DMPESI-induced surface passivation and reorganization, potentially involving halide exchange mechanisms previously reported for similar modifiers. Supplementary Note 3 provides further mechanistic insights,^11–13^ including valence band shifts (Figure S15) and its correlation with the observed improvements in *J*_SC_, *V*_OC_, and fill factor (FF) (Figure 1C), as well as their reduced losses under decreasing light intensity (Figure S16). These findings highlight the dual functionality of the DMPESI interlayer—enhancing PIM film morphology and providing effective surface passivation—both contributing to performance gains. While DMPESI has been recently applied to LHPs,^11^ this work demonstrates its effectiveness in the compositionally and electronically distinct pnictogen-based PIMs under indoor illumination.

The champion device achieved a record indoor PCE of 10.11% for wide-bandgap Sb- and Bi-based halide PIMs, with a *J*_SC_ of 73.8 μA cm^–2^, *V*_OC_ of 0.65 V, and FF of 69.3%, and a corresponding stabilized PCE of 9.6% (Figure 1E,F and Table 1). A *J*_SC_ mismatch of less than 10% was observed (Figure S17, Table S4), confirming good measurement consistency. The device also demonstrated reliable performance across various WLED color temperatures (Table S5) and light intensities from 1000 to 50 lux, with minimal efficiency loss (Figure 1E,F and Table 1). This makes these IPVs suitable also for realistic low-light conditions commonly found in indoor environments, with illumination levels as low as 100–50 lux.^15^ Under 1-sun illumination, our best devices achieved 4.4–4.6% PCE (4.34% stabilized), representing the highest reported to date for wide-bandgap (> 1.7 eV) halide PIMs (Figures S18–S19).

**Table 1:** Performance Metrics of the Champion CsMAFA-Sb:Bi Device (10 mm^2^ area) under Varying Illuminance Levels of WLED Lighting (6500 K Color Temperature)^a^

illuminance (lux)	input power density (μW cm^–2^)	PCE (%)	stabilized PCE (%)	power density output (μW cm^–2^)
1000	329	10.11	9.6	31.6
200	69	8.69	8.0	5.5
50	17	7.94	6.3	1.1

a The illumination levels selected (50, 200, and 1000 lux) follow the IEC TS 62607-7-2:2023 guidelines for standardized indoor PV characterization.^14^.

Achieving over 10% indoor PCE with stable operation at 1000 lux (Figure S20) establishes CsMAFA-Sb:Bi as a promising low-toxicity absorber for next-generation IPVs, with clear potential for further performance enhancement through film optimization and device engineering. The dual-function DMPESI interlayer improves microstructure through surface reconstruction and reduces interfacial carrier trapping, addressing long-standing challenges of PIM film uniformity. These performance gains underscore the importance of surface engineering in advancing PIM-based IPVs. Continued development of tailored passivation strategies, along with the adaptation of proven passivators from established technologies and thoughtful compositional engineering, could potentially boost indoor PCEs beyond 20–30%. Combined with application-relevant output levels (Supplementary Note 4) and integration considerations such as voltage stacking and power management ICs (Supplementary Note 5), these advancements could bring the vision of sustainable, high-efficiency ambient energy harvesting significantly closer to reality.

## Supplementary Material





## Notes

Experimental Section (including the indoor photovoltaics reporting checklist); Figures illustrating *J–V* curves of champion devices employing PPDT2FBT as the HTL under 1-Sun illumination (Figure S1); Performance optimization through device engineering (Figures S2–S6 and S8); Cross-sectional scanning electron microscopy (SEM) image of an optimized CsMAFA-Sb:Bi IPV device (Figure S7); Statistical distribution of unpassivated and DMPESI-passivated devices under 200 and 50 lux WLED illumination (Figure S9 and S10); XPS analysis (Figures S11–S13 and S15); SEM images of unpassivated and DMPESI-passivated CsMAFA-Sb:Bi films (Figure S14); Light-intensity dependence study (Figure S16); EQE spectra and corresponding integrated *J*_SC_ profiles at three illumination levels (Figure S17); *J–V* curves and SPOs of champion devices under 1-Sun illumination (Figure S18); Performance benchmark comparison of wide-bandgap Sb-, Bi-, and Sb-bibased halide PIMs (Figure S19); MPP tracking under indoor lighting for early stage operational stability assessment (Figure S20); Supporting tables (Tables S1–S5); and Supplementary Notes 1–5 (PDF)
Calibrated spectral irradiance data (1000 lux, WLED, 6500 K) measured using a spectroradiometer. The data were also converted to photon flux density for EQE-based integrated current calculations (XLSX)
