# Supplementary material for: Surpassing the 10% Efficiency Threshold in Perovskite-Inspired Indoor Photovoltaics
Source: ACS Energy Lett. 2025 Jun 25;10(7):3415–8. doi: 10.1021/acsenergylett.5c01472 (PMC12261317; doi:10.1021/acsenergylett.5c01472)
Supplement: Supplementary file 1 [file nz5c01472_si_001.pdf]

Surpassing the 10% Efficiency Threshold in Perovskite-Inspired Indoor Photovoltaics

Noora Lamminen, Jussi Lahtinen, Mokurala Krishnaiah, Joshua Karlsson, Milan Saju, G. Krishnamurthy Grandhi, Paola Vivo**

Hybrid Solar Cells, Faculty of Engineering and Natural Sciences, Tampere University, P. O. Box 541, Tampere, FI-33014, Finland.

E-mail: paola.vivo@tuni.fi; murthy.grandhi@tuni.fi

Table of Contents

Experimental Section 5

Indoor photovoltaics reporting checklist 8

Figure S1. Current density–voltage (*J–V*) curves of the champion device with the architecture FTO/c-TiO_2/m-TiO_2+CsMAFA-Sb:Bi/DMPESI/PPDT2FBT/Au, fabricated using a 60 mg/mL m-TiO_2 precursor solution (top panel), measured under 1-Sun illumination. The arrows indicate the scan direction. The corresponding stabilized power conversion efficiency (PCE) of approximately 4% was determined via maximum power point tracking (bottom panel). 10

Figure S2. Variation of photovoltaic parameters for FTO/c-TiO_2/m-TiO_2+CsMAFA-Sb:Bi/DMPESI/PPDT2FBT/Au devices as a function of m-TiO_2 precursor concentration under 1000 lux 6500 K WLED illumination. 11

Figure S3. UV-visible absorption spectra of CsMAFA-Sb:Bi films deposited on m-TiO_2 processed with varying precursor concentrations. 12

Figure S4. Variation of photovoltaic parameters for FTO/c-TiO_2/m-TiO_2+CsMAFA-Sb:Bi/DMPESI/PPDT2FBT/Au devices as a function of m-TiO_2 precursor concentration under 1-Sun illumination. 13

Figure S5. Photovoltaic parameters of FTO/c-TiO_2/m-TiO_2+CsMAFA-Sb:Bi/DMPESI/HTL/Au devices under 1-Sun illumination, using a 60 mg/mL m-TiO_2 precursor solution. The hole transport layers (HTLs) tested include PPDT2FBT, Spiro-OMeTAD, and PTAA. 14

Figure S6. Photovoltaic performance of FTO/c-TiO_2/m-TiO_2+CsMAFA-Sb:Bi/DMPESI/Spiro-OMeTAD/Au devices under 1000 lux, 6500 K WLED illumination, comparing m-TiO_2 precursor concentrations of 60 and 90 mg/mL. The stabilized PCE of 8.75% for the best-performing device with 90 mg/mL m-TiO_2 is shown at the bottom of the figure. 15

Figure S7. Cross-sectional SEM image of the optimized CsMAFA-Sb:Bi indoor photovoltaic device with the structure FTO/c-TiO_2/m-TiO_2/CsMAFA-Sb:Bi/DMPESI/Spiro-OMeTAD/Au. 16

Supplementary Note 1: Optimization process for maximizing IPV performance of CsMAFA-Sb:Bi 16

Figure S8. Systematic optimization of the mesoporous TiO_2 (m-TiO_2) scaffold and CsMAFA-Sb:Bi absorber precursor concentration for enhanced indoor photovoltaic (IPV) performance under 1000 lux, 6500 K WLED illumination, using Spiro-OMeTAD as the HTL. Statistical distributions of PCE, J_{SC}, V_{OC}, FF, and *J-V* hysteresis for devices with varying absorber concentrations (1x, 1.25x, and 1.5x) at a fixed m-TiO_2 precursor concentration of 90 mg/mL, and for the optimized combination of 1.25x absorber with 120 mg/mL m-TiO_2. 18

Figure S9. Distributions of PCE, J_{SC}, V_{OC}, and FF for unpassivated ('Ref') and DMPESI-passivated ('DMPESI') devices using Spiro-OMeTAD as the HTL, under 200 lux WLED illumination (6500 K). 19

Figure S10. Distributions of PCE, J_{SC}, V_{OC}, and FF for unpassivated ('Ref') and DMPESI-passivated ('DMPESI') devices using Spiro-OMeTAD as the HTL, under 50 lux WLED illumination (6500 K). 19

Supplementary Note 2: XPS analysis 20

Figure S11. Survey scan and the high resolution of XPS spectra of various elements of the unpassivated CsMAFA-Sb:Bi sample. 20

Figure S12. Survey scan and the high-resolution core levels of XPS spectra of various elements of the DMPESI-passivated CsMAFA-Sb:Bi sample. 21

Figure S13. The comparison of the high-resolution core levels of XPS spectra of I3d, Bi 4f, Sb 3d, and Cs3d of the unpassivated (reference) and DMPESI-passivated CsMAFA-Sb:Bi samples. Refer to the corresponding Table S2 for detailed peak shift values resulting from DMPESI surface passivation. 22

Figure S14. Scanning electron microscopy images of unpassivated (left) and DMPESI-passivated (right) films on FTO/c-TiO_2/m-TiO_2 substates. The DMPESI surface treatment effectively reduced the frequency of voids in the CsMAFA-Sb:Bi layer, resulting in a more uniform and compact morphology. 22

Supplementary Note 3: Mechanistic Insights into DMPESI-Induced Surface Modification 23

Figure S15. Valence band spectra of unpassivated and DMPESI-passivated CsMAFA-Sb:Bi samples, showing both the raw (left) and differentiated (right) data. After surface passivation, the valence band shifts toward lower binding energy. 24

Figure S16. Indoor light-intensity-dependent photovoltaic characteristics (50–1000 lux) of unpassivated and DMPESI-passivated CsMAFA-Sb:Bi devices. (A) Fill factor (FF) trends as a function of light intensity. Semi-logarithmic plots of V_{OC} vs. light intensity for (B) unpassivated and (C) DMPESI-passivated CsMAFA-Sb:Bi devices. The lowered FF losses and reduced ideality factor observed in the passivated devices indicate suppressed defect-related recombination, attributed to effective passivation of the CsMAFA-Sb:Bi layer. 25

Figure S17. (A) External quantum efficiency (EQE) spectrum of the CsMAFA-Sb:Bi champion device with the architecture FTO/c-TiO_2/m-TiO_2+CsMAFA-Sb:Bi/DMPESI/Spiro-OMeTAD/Au. (B) Photon flux density spectra of the 6500 K white LED (WLED) at illumination levels of 1000, 200, and 50 lux, along with the corresponding integrated short-circuit current density (J_{SC}) profiles. 26

Figure S18. Current density–voltage (*J–V*) curves of the champion devices with the architecture FTO/c-TiO_2/m-TiO_2+CsMAFA-Sb:Bi/DMPESI/Spiro-OMeTAD/Au, fabricated using m-TiO_2 precursor solutions at concentrations of 90 mg/mL (top panel) and 120 mg/mL (bottom panel), measured under 1-Sun illumination. Arrows indicate the *J–V* scan direction. Insets show the corresponding device parameters and stabilized PCE curves, obtained via maximum power point tracking. The J_{SC} values derived from integrating the EQE spectra over the 1-sun spectrum were 8.4 mA/cm^2 (J_{SC} mismatch: 5.3%) for the 90 mg/mL device, and 8.5 mA/cm^2 (J_{SC} mismatch: 4.2%) for the 120 mg/mL device. 27

Figure S19. Evolution of power conversion efficiency (PCE) over time for wide-bandgap (>1.7 eV) Sb-, Bi-, and Sb–Bi-based halide perovskite-inspired materials (PIMs) under 1-Sun illumination, plotted by year of publication. The comparison includes only devices where the

PIM serves as the sole light absorber and highlights the performance of our CsMAFA-Sb:Bi device relative to previously reported values. 28

Figure S20. Normalized power conversion efficiency (PCE) of a CsMAFA-Sb:Bi device measured under continuous 1000 lux WLED illumination of color temperatures of 4000 K (A) and 6500 K (B), in a nitrogen atmosphere, using maximum power point (MPP) tracking. T_{80} refers to the time required for the device's PCE to drop to 80% of its initial value (serves as a standard metric for operational stability). 29

Supplementary Note 4: Power Output Relevance to IoT Applications 30

Supplementary Note 5: Practical Integration Considerations for IoT Applications 30

Supporting Tables 32

Table S1. Average photovoltaic parameters (from 20 devices per group) for unpassivated and DMPESI-passivated CsMAFA-Sb:Bi devices with the architecture FTO/c-TiO_2/m-TiO_2+CsMAFA-Sb:Bi/DMPESI/Spiro-OMeTAD/Au under 1000 lux WLED illumination (6500 K). The highest value for each parameter is shown in parentheses. 32

Table S2. Binding energy values of various elements in unpassivated and DMPESI-passivated CsMAFA-Sb:Bi films, along with the corresponding shifts (ΔE) in binding energy. 32

Table S3. Grain size analysis and roughness parameters for unpassivated and DMPESI-passivated CsMAFA-Sb:Bi film samples based on AFM measurements. Here the roughness was measured from a 128 x 512 section of the sample in the centre of the frame. RMS = root mean square. The mean size is calculated by averaging the equivalent square sides. Its square is not, in general, equal to the mean area. 33

Table S4. Comparison of short-circuit current densities (J_{SC}) derived from reverse and forward J–V scans and EQE integration under 6500 K white LED (WLED) illumination at 1000, 200, and 50 lux. J_{SC} mismatch percentages are calculated relative to EQE-derived J_{SC} values, and the average mismatch provides an overall measure of consistency across methods. 33

Table S5. The photovoltaic performance metrics of the champion CsMAFA-Sb:Bi device measured under 1000 lux WLED illumination across different color temperatures. 33

REFERENCES 34

Experimental Section

Materials

Antimony iodide (SbI_3, 99.999%, Fisher), cesium iodide (CsI, 99.999%, ABCR), bismuth iodide (BiI_3, 99.998%, Sigma-Aldrich), methylammonium chloride (MACl, 99.99%, Greatcell Solar Materials), formamidium iodide (FAI, 99.99%, Greatcell Solar Materials), dimethyl sulfoxide (DMSO, anhydrous, ≥99.9%, Sigma-Aldrich), *N,N*-dimethylformamide (DMF, anhydrous, 99.8%, Sigma-Aldrich), 2-propanol (anhydrous, 99.5%, Sigma-Aldrich), chlorobenzene (CB, anhydrous, 99.8%, Sigma-Aldrich), titanium diisopropoxide bis(acetylacetonate) 75 wt% in 2-propanol (Sigma-Aldrich), 30 NR-D Titania Paste (Greatcell Solar Materials), dimethylphenethylsulfonium iodide (DMPESI, >98%, TCI), 2,2',7,7'-Tetrakis(N,N-di-p-methoxyphenylamino)-9,9'-spirobifluorene (Spiro-OMeTAD, ≥99.5%, Luminescence Technology Corp), poly[(2,5-bis(2-hexyldecyloxy)phenylene)-alt-(5,6-difluoro-4,7-di(thiophen-2-yl)benzo[c]-[1,2,5]thiadiazole)] (PPDT2FBT, 70–100 kg mol^{-1}, Brillian Matters), acetonitrile (anhydrous, 99.9%, Sigma-Aldrich), 4-*tert*-Butylpyridine (*t*BP, 98%, Sigma-Aldrich), lithium bis(trifluoromethanesulfonyl)imide (Li-TFSI, 99.95%, Sigma-Aldrich), tris[2-(1H-pyrazol-1-yl)−4-tert- butylpyridine]cobalt(III)tri[bis-(trifluoromethane) sulfonimide] (FK209 Co(III), >98%, Dyenamo), poly[bis(4-phenyl)(2,4,6-trimethylphenyl)amine] (PTAA ,Sigma-Aldrich), toluene (anhydrous, 99.8%, Sigma-Aldrich). All reagents and solvents were used as received without additional purification.

Precursor solution preparation

All precursor solutions were fabricated inside a N_2 filled glovebox. The CsMAFA-Sb:Bi precursor was fabricated from two separate CsMAFA-Sb and CsMAFA-Bi precursors by mixing them in a 90:10 ratio. The solution preparation is described in Lamminen et al.[1] The 1.25× and 1.5× precursors were fabricated from single solutions. The 1.25× precursor solution consisted of SbI_3, CsI, BiI_3, MACl and FAI in the molar ratio 0.9:1.2:0.1:1.5:0.3, with 150 mol% of MACl and 30 mol% of FAI compared to the combined amount of SbI_3 and BiI_3, at a concentration of 560 mg/mL. For 1000 µL of the 1.25× precursor solution the amounts were SbI_3 259.25 mg, CsI 178.75 mg, BiI_3 33.75 mg, MACl 58.13 mg, FAI 29.69 mg, DMF 500 µL, and DMSO 500 µL. The 1.5× precursor solution consisted of SbI_3, CsI, BiI_3, MACl and FAI in the same molar ratio at a concentration of 672 mg/mL. For 1000 µL of the 1.5× precursor solution the amounts were SbI_3 311.25 mg, CsI 214.50 mg, BiI_3 40.50 mg, MACl 69.75 mg, FAI 35.75 mg, DMF 500 µL, and DMSO 500 µL. The precursors were stirred 12-24 hours before use.

Solar cell fabrication

Pre-patterned fluorine-doped tin oxide (FTO) coated substrates (TEC15, 2.2 mm thick, OPV Tech) were cleaned by brushing with 2% Mucasol solution, rinsed with ultra-pure water and subsequently sonicated in ultra-pure water, acetone, and 2-propanol for 15 minutes in each solvent. The substrates were dried with a N_2 flow.

A compact titanium dioxide (TiO_2) layer was deposited on the clean substrates *via* spray pyrolysis at 450 °C. The precursor was fabricated by diluting 1.15 mL of titanium diisopropoxide bis(acetylacetonate) 75 wt% in 2-propanol with 4.95 mL of 2-propanol. The layer was deposited by 13 spray cycles with 20 s between each spray cycle. The substrates were then annealed at 450 °C for 45 minutes. The mesoporous TiO_2 layer was deposited by spin-coating from a solution of 30 NR-D Titania Paste in ethanol. Different concentrations of the solution from 30 to 150 mg/mL were tested. Per substrate, 80 μl of the solution was spin-coated at 4000 rpm for 10 s (2000 rpm s^{-1} acceleration), followed by annealing at 100 °C for few minutes, and calcination at 450 °C for 30 min with a 45 min ramp time. The substrates were then cooled down to 200 °C and transferred to a N_2 filled glove box for PIM deposition.

The PIM layers were deposited by pipetting 60 μL of the precursor on the substrate, followed by spin-coating at 3000 rpm for 30 s (acceleration within 5 s). When there was 10 s of the spin-coating program left, 150 μL of 2-propanol antisolvent was added. The films were subsequently annealed at 130 °C for 10 min. 100 μL of DMPESI (5 mg/mL in chloroform) interfacial modifier was spin coated on the PIM layer dynamically with 4000 rpm 20 s spin-coating program. The films were annealed at 100 °C for 10 min.

Several different HTMs were tested in this work. PPDT2FBT (10 mg/mL in 1,2-dichlorobenzene, dissolved by stirring at 60 °C overnight) was deposited by dynamically spin-coating 70 μL with 2000 rpm 30 s spin-coating program. The films were annealed at 80 °C for 10 min. Spiro-OMeTAD (36.2 mg/mL in chlorobenzene) was doped with *t*BP, Li-TFSI (520 mg/mL in acetonitrile), and FK209 Co(III) (300 mg/mL in acetonitrile). The typical amounts for 1000 μL of the Spiro-OMeTAD solution were 14.4 μL, 8.8 μL, and 14.5 μL, respectively. The doped Spiro-OMeTAD solution was dynamically spin-coated using an 1800 rpm 30 s program, dispensing 80 μL per substrate. PTAA (10 mg/mL in toluene) was spin-coated dynamically with 5000 rpm 30 s spin-coating program, dispensing 80 μL per substrate. Finally, the solar cells were completed by thermally evaporating 100 nm thick gold contacts under 6×10^{-6} mbar vacuum.

Photovoltaic device measurements

The current-voltage (I-V) characteristics of the cells were measured under 1-Sun AM 1.5G conditions under an A++A+A SINUS-70 LED solar simulator (Wavelabs). The I-V and stabilized power output (SPO), obtained via maximum power point tracking, measurements were recorded with a Litos Lite parallel *J-V* system (Fluxim) in ambient conditions with 50 mVs^{-1} scan rate. 10 mm^2 aperture masks were used to define the device area.

The **indoor I-V** characteristics and SPOs of the cells were measured under different lighting levels and color temperatures using an adjustable HUE WLED bulb (Philips, controlled by Philips Hue Essential App) in the ambient environment in a black box to minimize reflections. The power density and color temperature of the bulb were measured with a BTS256-WF spectral light meter (Gigahertz-Optik). The scans were recorded with a Keithley 4250 source-monitor unit using a 4-wire setup with 50 mVs^{-1} scan rate. 400 µm thick 10 mm^2 aperture masks were used to define the device area. In maximum power point tracking the unencapsulated devices were kept under N_2 flow.

The **external quantum efficiency** of the cells was measured in dark conditions with a QuantX-300 (Newport) device. The integrated J_{SC} values were estimated by integrating the EQE spectra over the incident photon flux of the AM 1.5G (1-Sun) or the different indoor spectra recorded with the BTS256-WF spectral light meter (spectroradiometer).

X-ray photoelectron spectroscopy (XPS) measurements were performed using an ultrahigh vacuum (UHV) system. The perovskite films were deposited onto indium tin oxide (ITO)-coated glass substrates and left to degas overnight in a vacuum desiccator prior to introduction into the UHV chamber. XPS spectra were acquired using non-monochromatized Al $K\alpha_{1,2}$ radiation (photon energy = 1486.6 eV), produced by a twin anode X-ray source (8025 Twin Anode, V. G. Microtech), in conjunction with a hemispherical electron energy analyzer (CLAM4 MCD LNo5, V. G. Microtech). Data acquisition, processing, and analysis were performed using CasaXPS version 2.3.25 PR1.0. Peak fitting was carried out after Shirley background subtraction, employing a Gaussian–Lorentzian function to approximate the line shapes of the fitted components. Calibration of the spectra was achieved by setting the C 1s (C–C) peak to 284.8 eV.

Atomic force microscopy measurements were obtained with a Bruker Dimension Icon instrument, using the proprietary PeakForce tapping imaging mode. The imaging tip used was "SCANASSYT-AIR". The lift height was set to 150 nm and the measurement system allowed to optimise the probe force setpoint automatically (typically between 1 and 4 nN). Images were obtained at 512 x 512 pixel resolution using a scan speed of 0.5 Hz. All images were analysed using the Gwyddion software suite

(version 2.64).[2] For the purposes of presenting visually clear images, the AFM micrographs were flattened and scan defects removed.

Scanning electron microscopy (SEM) top-view images of DMPESI-passivated and unpassivated CsMAFA-Sb:Bi films on FTO/c-TiO_2/m-TiO_2 substrates were taken with a field emission scanning electron microscope (Zeiss Ultra Plus). The films were coated with 5 nm of carbon before imaging. The operation was at 3 kV acceleration voltage with InLens mode.

Indoor photovoltaics reporting checklist

Experimental Aspect	Detailed Description
**Indoor Light Source and its Calibration**	✓ White LED (Philips Hue Starter Kit E27-1100) emitting in the wavelength range ~400–750 nm.<br>✓ Diffuse illumination setup with LED mounted above the device; horizontal aperture between the source and device area minimizes stray reflections and controls illumination geometry.<br>✓ BTS256-WF spectroradiometer with a cosine-corrected diffuser positioned at device height to accurately measure illuminance, irradiance, and spectral distribution simultaneously, ensuring Lambert's cosine law compliance.<br>✓ No reference cell was used for calibration.<br>✓ Light source stabilizes within ~5 min after activation, demonstrating stable illuminance and irradiance over several days.<br>✓ Raw spectral irradiance data provided in supplementary material; additional data (irradiance, illuminance at different illuminances) provided in the main text.
**Illumination Conditions**	✓ Indoor photovoltaic performance evaluated at three illuminances (1000, 200, and 50 lux) as per IEC TS 62607-7-2:2023 recommendations.<br>✓ Illuminances adjusted using Philips Hue Essentials smartphone app.<br>✓ Performance further studied under multiple correlated color temperatures (CCTs: 6500 K, 5000 K, 4000 K, 2700 K).<br>✓ Device parameter variations are reported at different illuminances (50–1000 lux), assisting in analysis of linearity/non-linearity under varying indoor illumination conditions.

**IPV device Measurements**	✓ Power conversion efficiency (PCE) determined from current–voltage (I–V) curves measured between -0.2 and 0.8 V at a scan rate of 50 mV/s, performing reverse sweep followed by forward sweep.<br>✓ No device pre-conditioning was applied prior to I–V measurement, except a dwell time of 3 seconds.<br>✓ PCE values from reverse and forward sweeps: 10.11% and 9.44%, respectively.<br>✓ Stabilized PCE (9.6%) measured using a modified Perturb and Observe (P&O) Maximum power point (MPP) tracking algorithm, involving periodic voltage perturbations, a defined dwell time for transient effects stabilization, and average power calculation to determine MPP accurately.<br>✓ Device active area: 12.25 mm^2, achieved through laser etching of a 2.5 × 2.5 cm^2 substrate and subsequent gold mask evaporation.<br>✓ Aperture (metal mask) area: 10 mm^2; thickness: 400 nm—without using the aperture mask, photocurrent (J_{SC}) was significantly overestimated (by a factor of 2–4).
**Measurement Environment and Stability Testing**	✓ Current–voltage (I–V) measurements conducted in ambient air without encapsulation.<br>✓ MPP tracking conducted under nitrogen atmosphere at ambient room temperature (20–22 °C) without additional temperature control. A modified Perturb and Observe (P&O) MPP tracking algorithm, involving periodic voltage perturbations, a defined dwell time for transient effects stabilization, and average power calculation was used to determine MPP accurately.<br>✓ Long-term stability tested continuously for >100 hours under 1000 lux, 4000 K WLED illumination in a nitrogen environment, performed at MPP conditions (similar to the ISOS-L stability protocol published for solar cell stability assessment)
**Device Data Reporting, Shunt Resistance, and EQE Characterization**	✓ Statistical analysis conducted on 15 devices, clearly reporting both champion (10.11%) and average (9.70%) PCE at 1000 lux, alongside statistical distributions of other device parameters (V_{OC}, J_{SC}, FF, PCE).<br>✓ Photovoltaic parameters reported: output power density, open-circuit voltage (V_{OC}), short-circuit current density (J_{SC}), fill factor (FF), and power conversion efficiency (PCE) for each illuminance (50, 200, 1000 lux).<br>✓ Shunt resistance values for champion devices:<br>DMPESI-passivated (90909 $\Omega \cdot cm^2$), unpassivated (78740 $\Omega \cdot cm^2$).<br>✓ External quantum efficiency (EQE) measured using Newport QuantX-300 spectroradiometer under zero-bias conditions and without additional bias lighting, across 350–850 nm spectral range at 10 nm increments—Integrated J_{SC} values (the corresponding J_{SC} mismatch are also reported) reported at each illuminance (1000, 200, 50 lux).

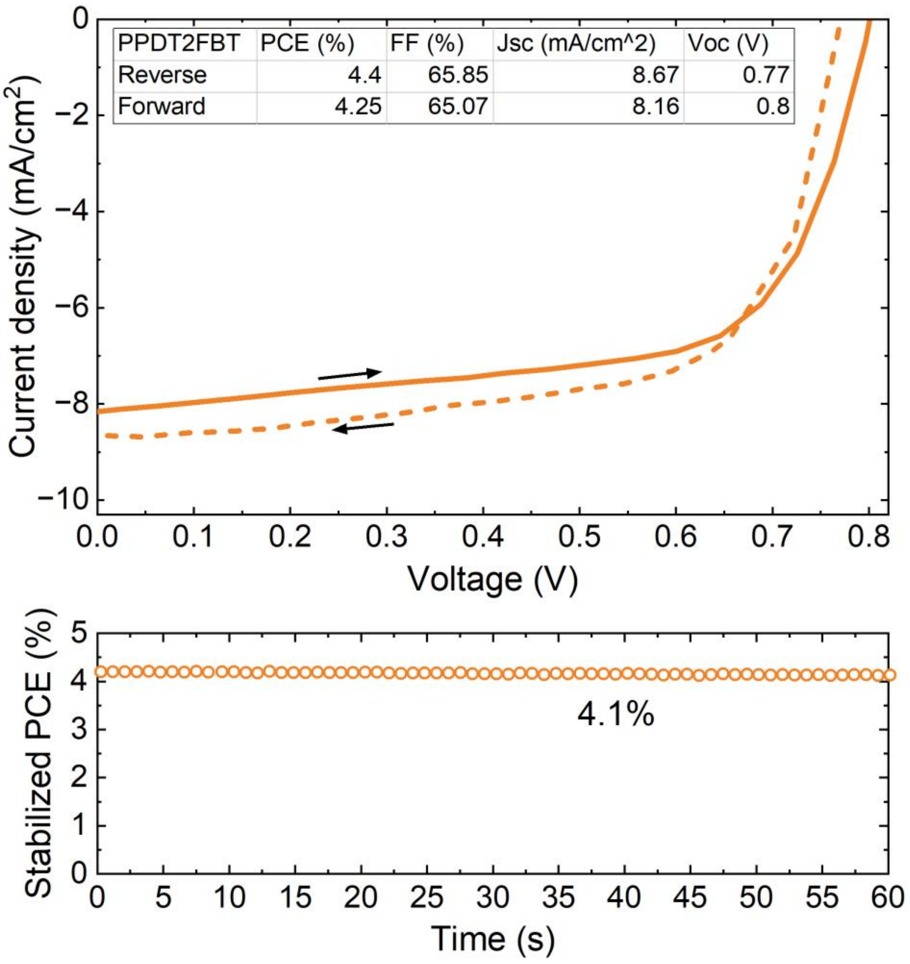

Figure S1. Current density–voltage (*J*–*V*) curves of the champion device with the architecture FTO/c-TiO_2/m-TiO_2+CsMAFA-Sb:Bi/DMPESI/PPDT2FBT/Au, fabricated using a 60 mg/mL m-TiO_2 precursor solution (top panel), measured under 1-Sun illumination. The arrows indicate the scan direction. The corresponding stabilized power conversion efficiency (PCE) of approximately 4% was determined via maximum power point tracking (bottom panel).

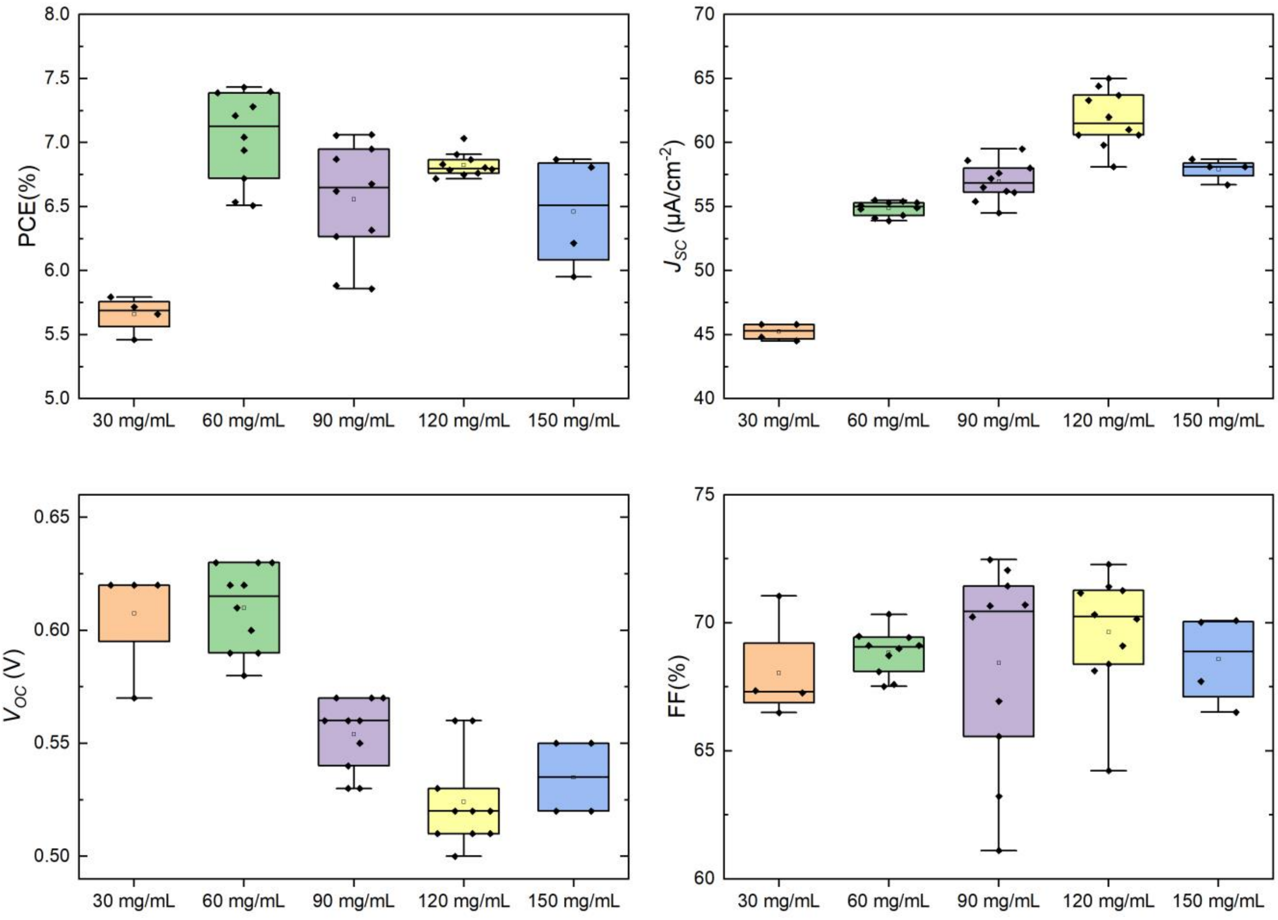

Figure S2. Variation of photovoltaic parameters for FTO/c-TiO_2/m-TiO_2+CsMAFA-Sb:Bi/DMPESI/PPDT2FBT/Au devices as a function of m-TiO_2 precursor concentration under 1000 lux 6500 K WLED illumination.

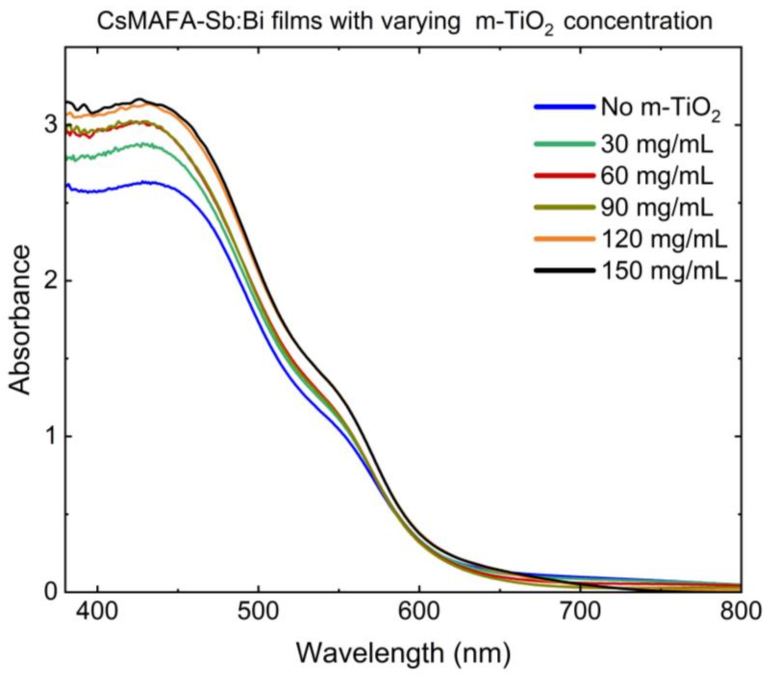

Figure S3. UV-visible absorption spectra of CsMAFA-Sb:Bi films deposited on m-TiO_2 processed with varying precursor concentrations.

UV–vis analysis showed that higher m-TiO_2 concentrations enhanced film thickness and light absorption, with ***Figures S2*** *and* ***S4*** *identifying the optimal concentration for peak performance with the PPDT2FBT HTL.*

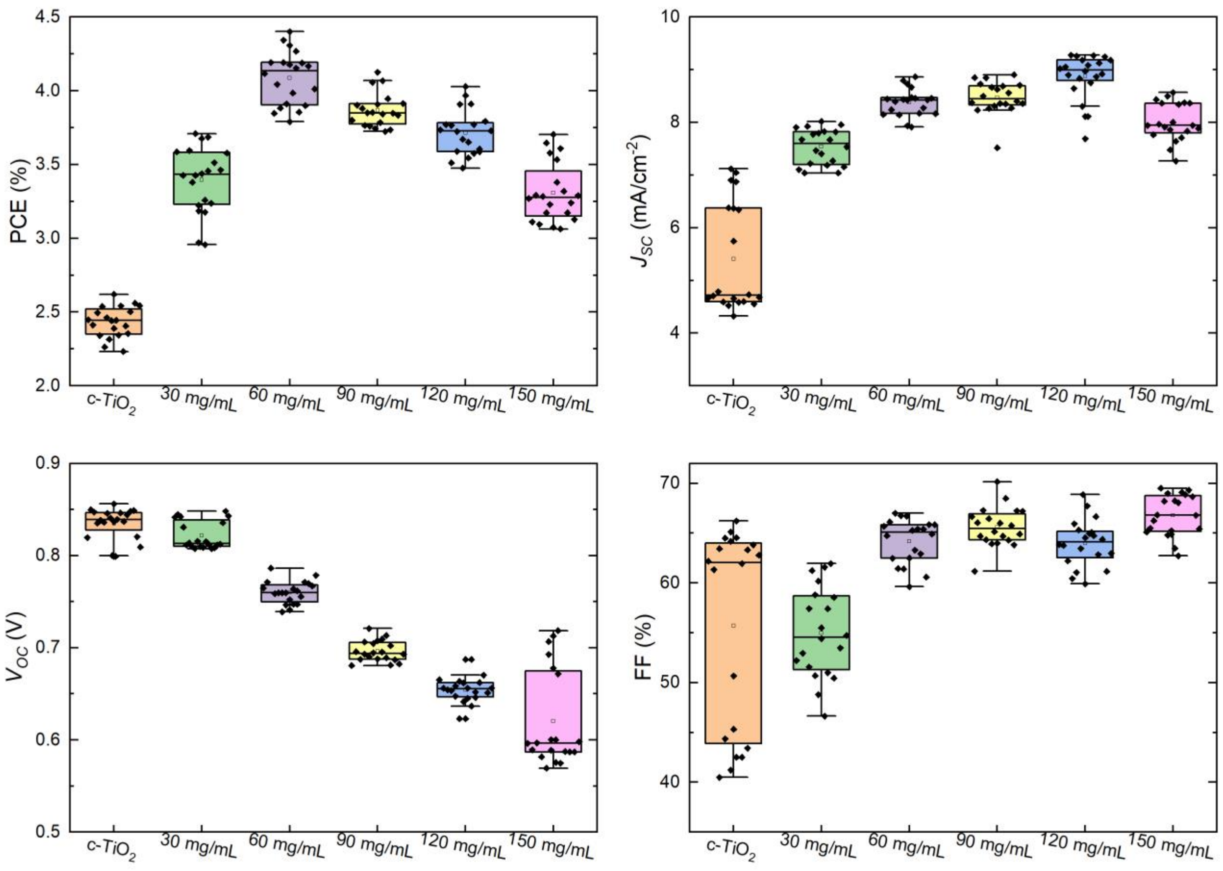

Figure S4. Variation of photovoltaic parameters for FTO/c-TiO_2/m-TiO_2+CsMAFA-Sb:Bi/DMPESI/PPDT2FBT/Au devices as a function of m-TiO_2 precursor concentration under 1-Sun illumination.

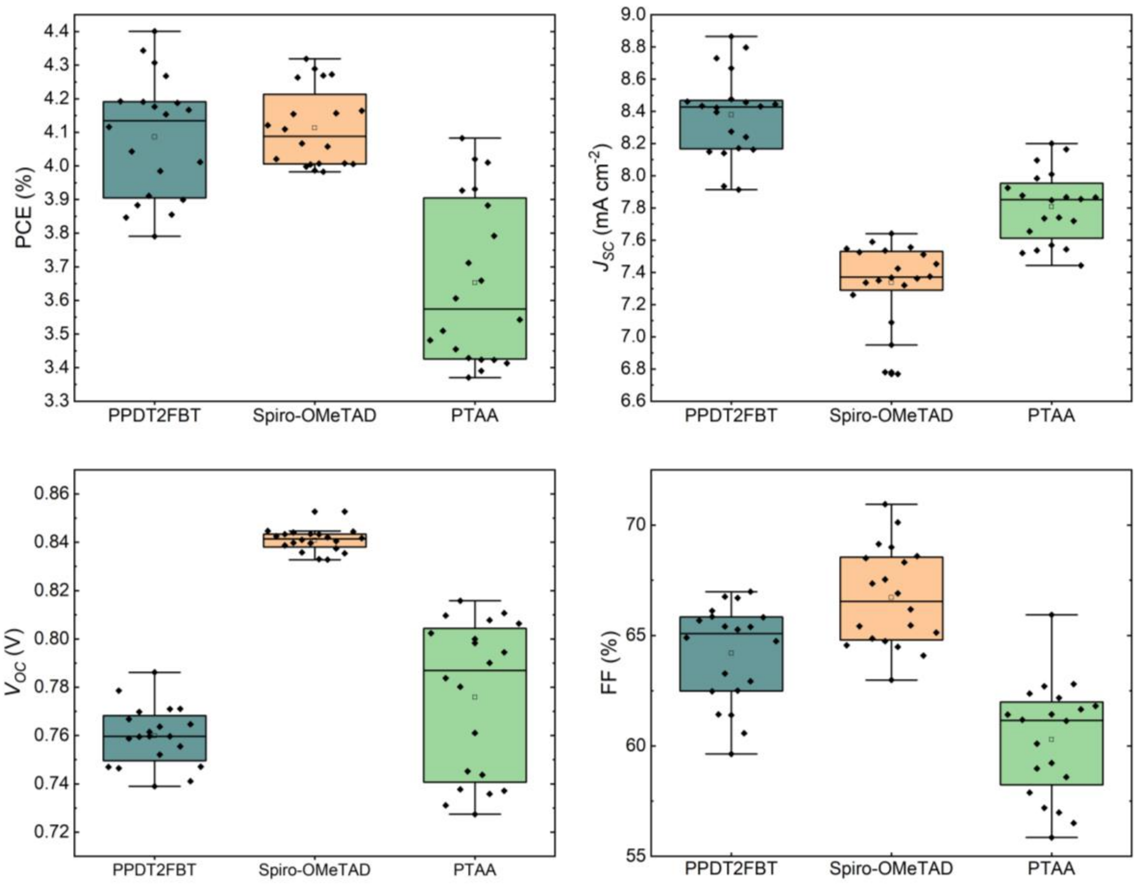

Figure S5. Photovoltaic parameters of FTO/c-TiO_2/m-TiO_2+CsMAFA-Sb:Bi/DMPESI/HTL/Au devices under 1-Sun illumination, using a 60 mg/mL m-TiO_2 precursor solution. The hole transport layers (HTLs) tested include PPDT2FBT, Spiro-OMeTAD, and PTAA.

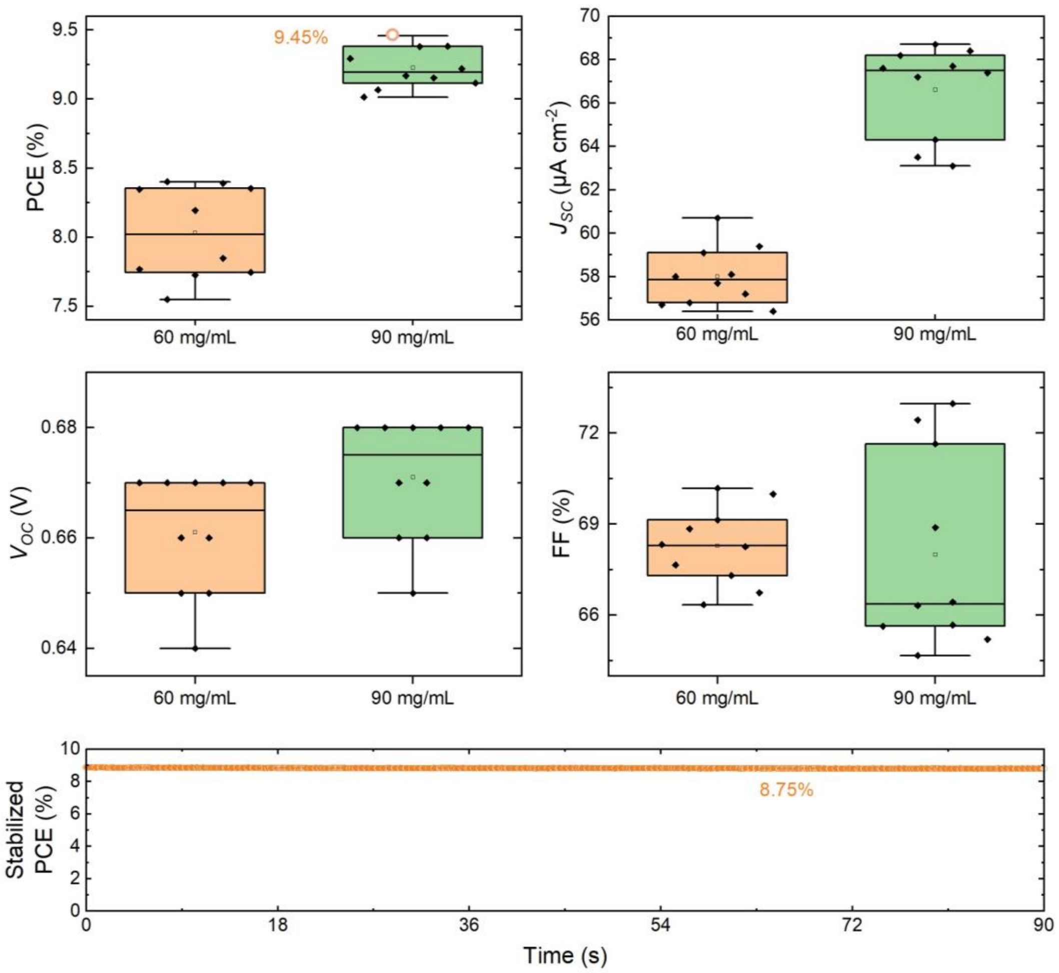

Figure S6. Photovoltaic performance of FTO/c-TiO_2/m-TiO_2+CsMAFA-Sb:Bi/DMPESI/Spiro-OMeTAD/Au devices under 1000 lux, 6500 K WLED illumination, comparing m-TiO_2 precursor concentrations of 60 and 90 mg/mL. The stabilized PCE of 8.75% for the best-performing device with 90 mg/mL m-TiO_2 is shown at the bottom of the figure.

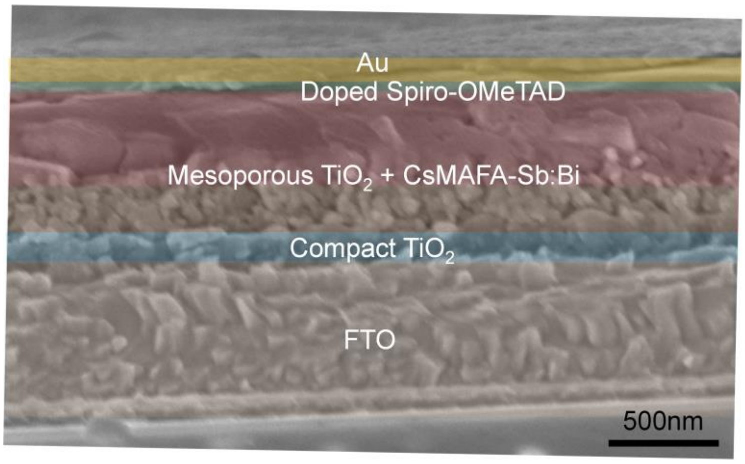

Figure S7. Cross-sectional SEM image of the optimized CsMAFA-Sb:Bi indoor photovoltaic device with the structure FTO/c-TiO_2/m-TiO_2/CsMAFA-Sb:Bi/DMPESI/Spiro-OMeTAD/Au. The apparent thicknesses of the absorber (~400 nm) and mesoporous TiO_2 layer (~200 nm) are indicated. These values correspond to the 120 mg mL^{-1} m-TiO_2 precursor concentration and reflect partial infiltration of the absorber into the mesoporous scaffold.

Supplementary Note 1: Optimization process for maximizing IPV performance of CsMAFA-Sb:Bi

After identifying Spiro-OMeTAD as the most effective HTL for maximizing the IPV performance of CsMAFA-Sb:Bi compared to PPDT2FBT, we systematically varied both the mesoporous TiO_2 (m-TiO_2) scaffold and Sb:Bi absorber layer concentrations, revealing a synergistic effect on device performance. Initially, increasing the m-TiO_2 precursor concentration from 60 to 90 mg/mL enhanced both J_{SC} and V_{OC}, indicating improved charge collection and reduced recombination at the electron transport interface (**Figure S6**). Subsequent optimization of the absorber concentration from 1× to 1.25× further boosted J_{SC}, likely due to increased light absorption from a thicker absorber layer. However, increasing the absorber concentration to 1.5× led to a decline in PCE, primarily due to a drop in J_{SC} and V_{OC}, suggesting increased bulk recombination (**Figure S8**).

Notably, retaining the optimized 1.25× absorber concentration while increasing the m-TiO_2 concentration to 120 mg/mL yielded the highest PCE (>10%), driven by a substantial improvement in J_{SC} (**Figure S8**). This enhancement likely arises from more efficient charge extraction due to better

pore infiltration of the thick perovskite-inspired absorber. This optimized device configuration also exhibited significantly reduced *J–V* hysteresis (**Figure S8**). These findings underscore the importance of co-optimizing mesoporous scaffold density and absorber layer thickness to balance light absorption, charge transport, and interfacial dynamics in PIM-based IPV devices.

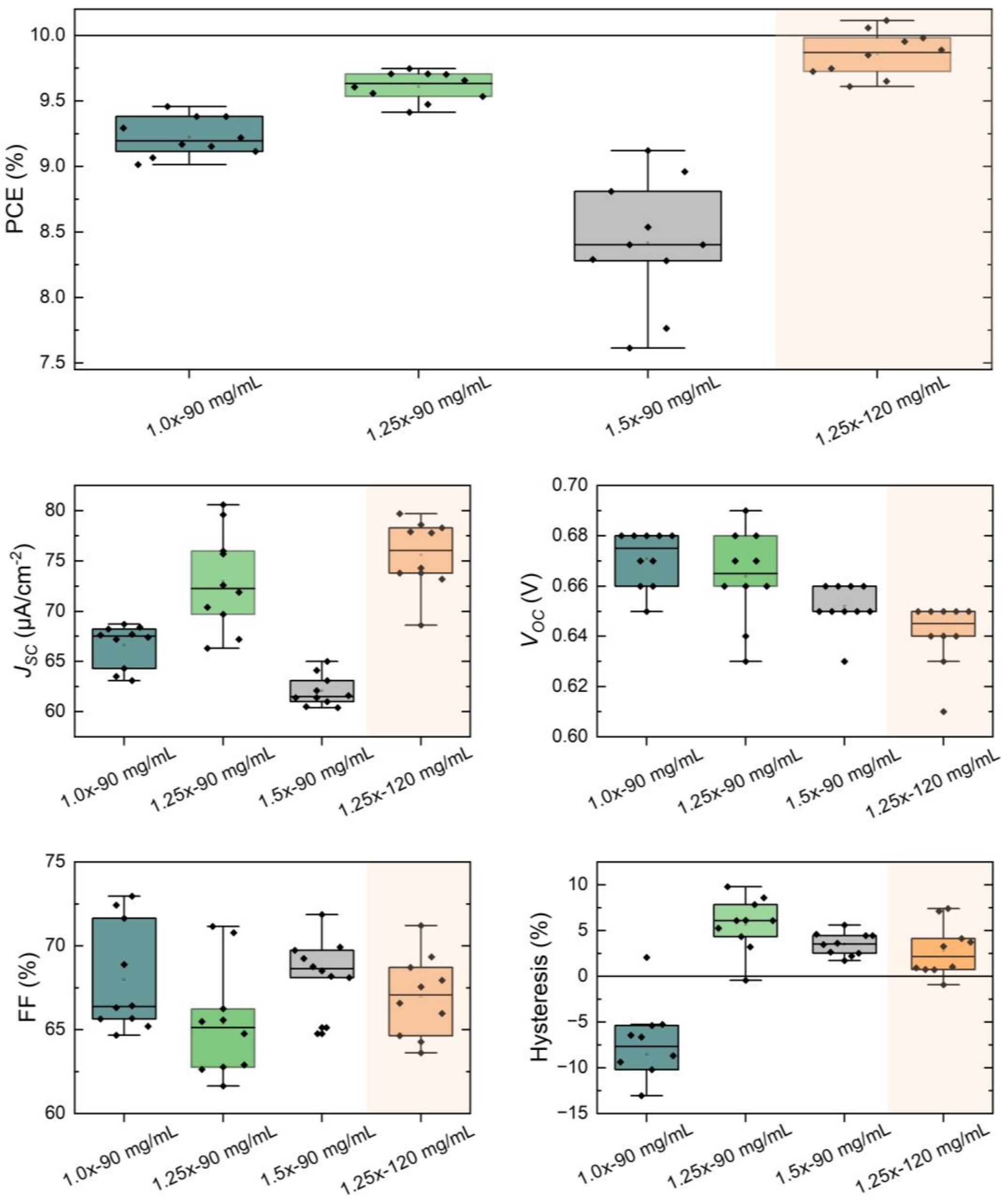

Figure S8. Systematic optimization of the mesoporous TiO_2 (m-TiO_2) scaffold and CsMAFA-Sb:Bi absorber precursor concentration for enhanced indoor photovoltaic (IPV) performance under 1000 lux, 6500 K WLED illumination, using Spiro-OMeTAD as the HTL. Statistical distributions of PCE, J_{SC}, V_{OC}, FF, and *J-V* hysteresis for devices with varying absorber concentrations (1x, 1.25x, and 1.5x) at a fixed m-TiO_2 precursor concentration of 90 mg/mL, and for the optimized combination of 1.25x absorber with 120 mg/mL m-TiO_2.

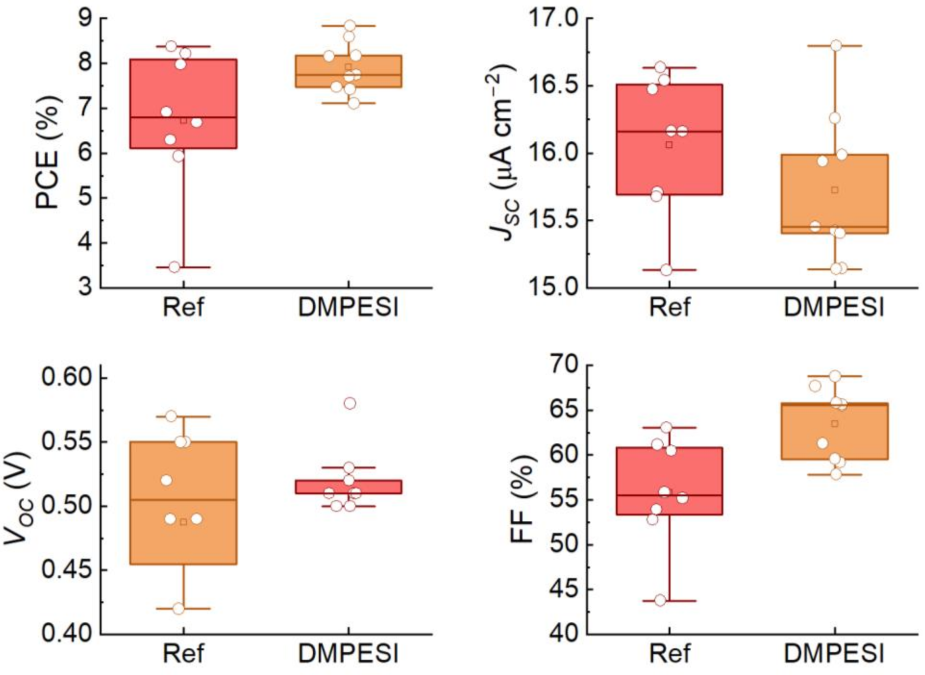

Figure S9. Distributions of PCE, J_{SC}, V_{OC}, and FF for unpassivated ('Ref') and DMPESI-passivated ('DMPESI') devices using Spiro-OMeTAD as the HTL, under 200 lux WLED illumination (6500 K).

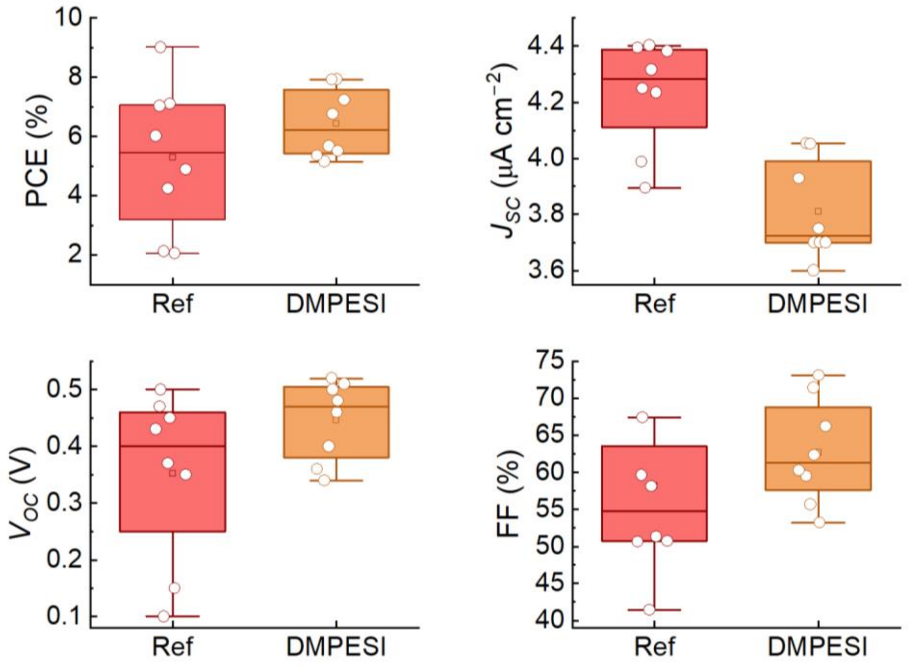

Figure S10. Distributions of PCE, J_{SC}, V_{OC}, and FF for unpassivated ('Ref') and DMPESI-passivated ('DMPESI') devices using Spiro-OMeTAD as the HTL, under 50 lux WLED illumination (6500 K).

Note: *Devices with DMPESI passivation show consistently higher average PCE,* V_{OC}*, and FF compared to control devices without DMPESI under both 200 and 50 lux illumination, confirming its beneficial effect across indoor light levels.*

Supplementary Note 2: XPS analysis

XPS survey spectra (**Figure S11** and **Figure S12**) of the unpassivated (reference) and DMPESI-passivated CsMAFA-Sb:Bi samples confirmed the expected presence of Cs, Sb, Bi, I, and Cl. The N 1s signal, attributed to the MA^+/FA^+ (N–C) groups in CsMAFA-Sb:Bi, was also detected at the surface. In the DMPESI-passivated sample, all elemental peaks were shifted to lower binding energies compared to the unpassivated sample (see **Figure S13** and **Table S2**), indicating strong interactions between the DMPESI molecules and the CsMAFA-Sb:Bi surface.

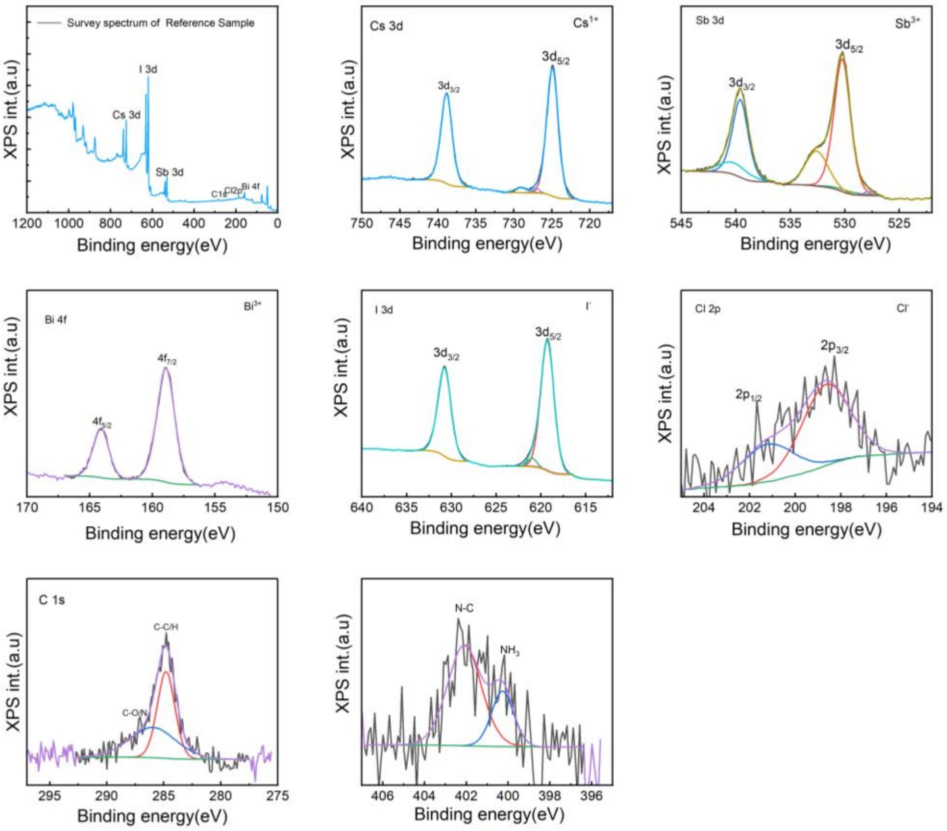

Figure S11. Survey scan and the high resolution of XPS spectra of various elements of the unpassivated CsMAFA-Sb:Bi sample.

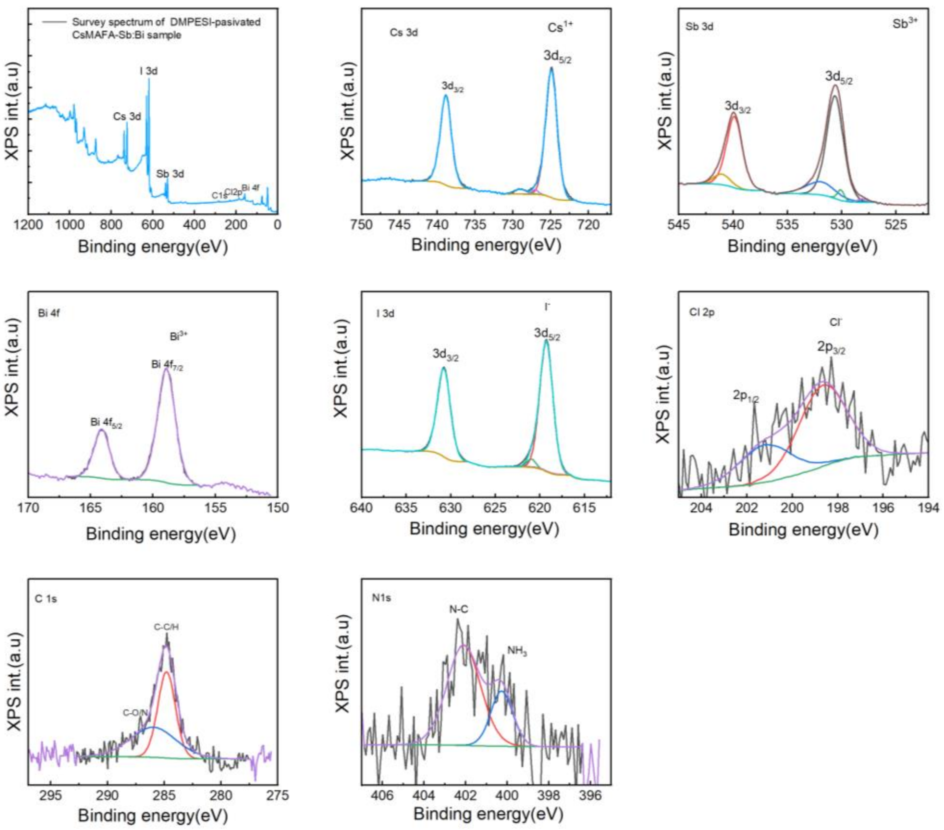

Figure S12. Survey scan and the high-resolution core levels of XPS spectra of various elements of the DMPESI-passivated CsMAFA-Sb:Bi sample.

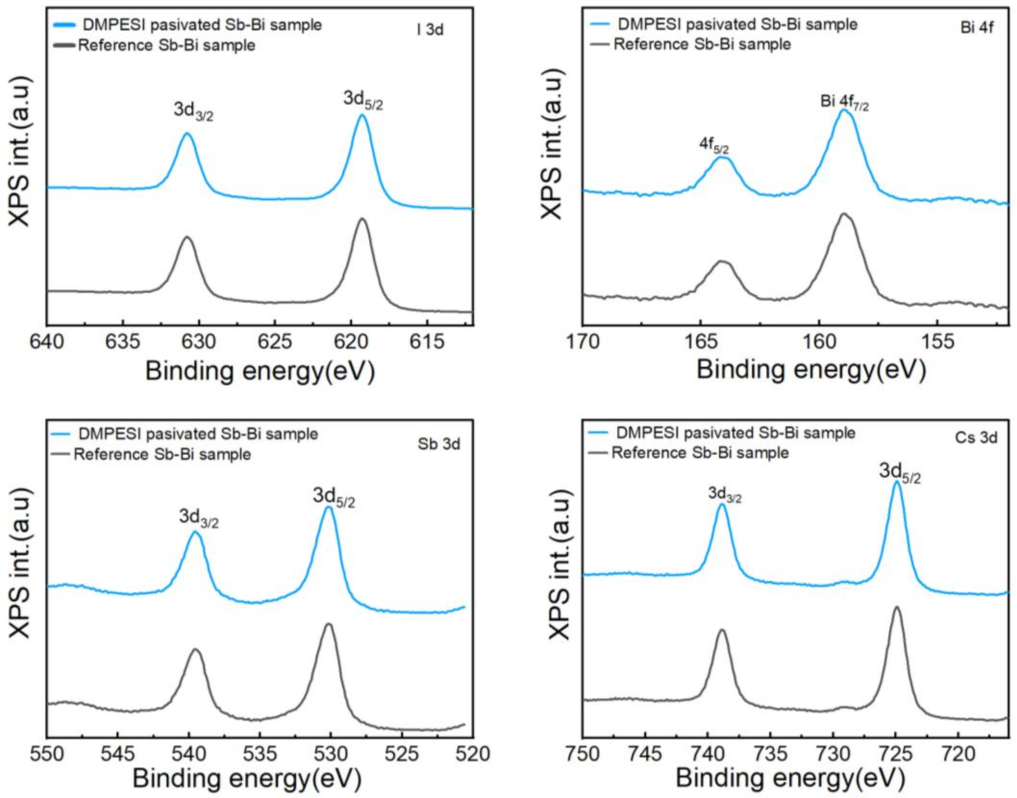

Figure S13. The comparison of the high-resolution core levels of XPS spectra of I3d, Bi 4f, Sb 3d, and Cs3d of the unpassivated (reference) and DMPESI-passivated CsMAFA-Sb:Bi samples. Refer to the corresponding Table S2 for detailed peak shift values resulting from DMPESI surface passivation.

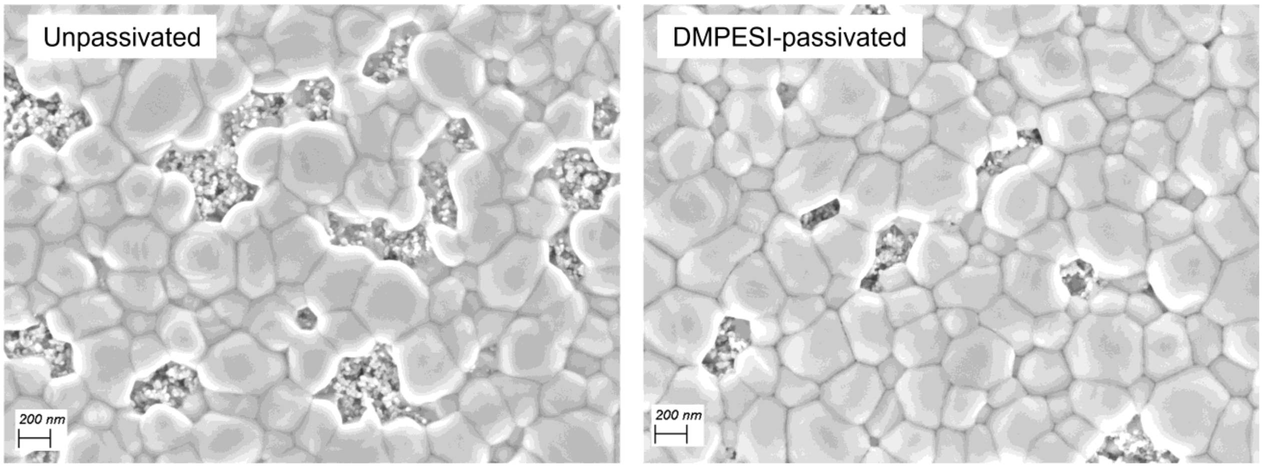

Figure S14. Scanning electron microscopy images of unpassivated (left) and DMPESI-passivated (right) films on FTO/c-TiO_2/m-TiO_2 substates. The DMPESI surface treatment effectively reduced the frequency of voids in the CsMAFA-Sb:Bi layer, resulting in a more uniform and compact morphology.

Supplementary Note 3: Mechanistic Insights into DMPESI-Induced Surface Modification

Our combined XPS, AFM, and SEM point towards DMPESI-induced surface passivation and reorganization of the Sb:Bi PIM layer. These effects likely result from thermally activated surface reorganization facilitated by the DMPESI treatment, attributed to its molecular composition and chemical functionality. This behavior aligns with recent findings where surface-applied molecular species, such as benzyl chloromethyl sulfide and DMPESI, induced in situ chemical reactions and surface reconstruction via halide incorporation in fully crystallized perovskites.[3–5] A similar halide exchange-induced surface reconstruction may also be operative in our Sb-Bi PIM, complementing the DMPESI-mediated passivation and morphological reorganization observed in our study. Notably, XPS measurements indicate that DMPESI chemically interacts with the PIM surface, suggesting that the passivation effect is not purely physical (e.g., surface coverage), as also supported by AFM data, but involves reactive modification of surface states. While our current data and prior literature support this interpretation, a more complete mechanistic understanding would benefit from future density functional theory (DFT) calculations.

Additionally, the valence band shift after DMPESI passivation (**Figure S15**) may suggest reduced surface defects and improved interfacial energy-level alignment.[6] This interpretation is further supported by the observed improvements in J_{SC}, V_{OC}, and fill factor (FF) (**Figure 1C** in main text). Moreover, the reduced losses in V_{OC} and FF with decreasing indoor light intensity in the passivated devices (**Figure S16**) provide additional evidence for the effectiveness of the passivation strategy.

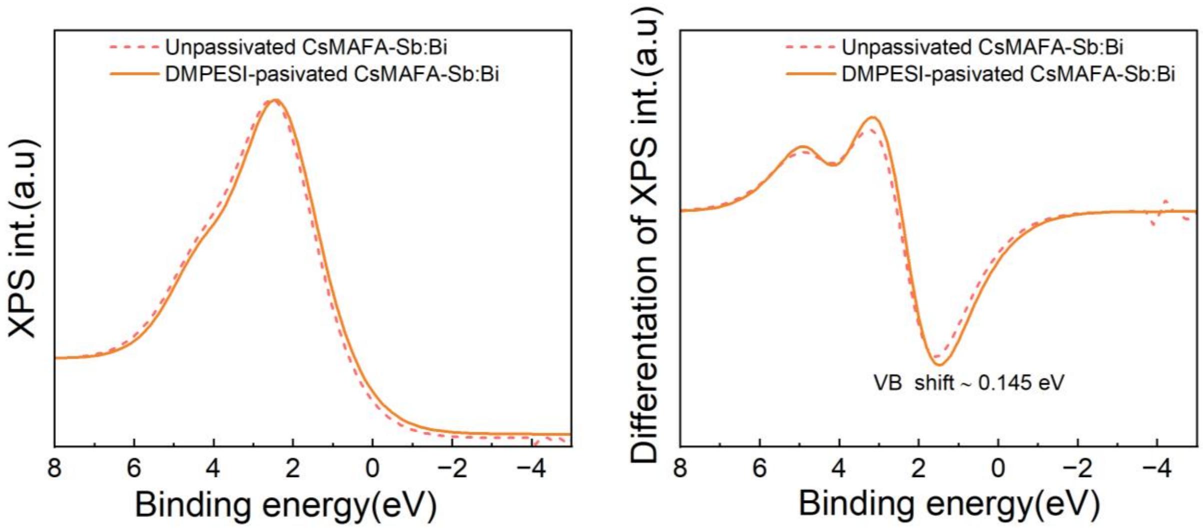

Figure S15. Valence band spectra of unpassivated and DMPESI-passivated CsMAFA-Sb:Bi samples, showing both the raw (left) and differentiated (right) data. After surface passivation, the valence band shifts toward lower binding energy.

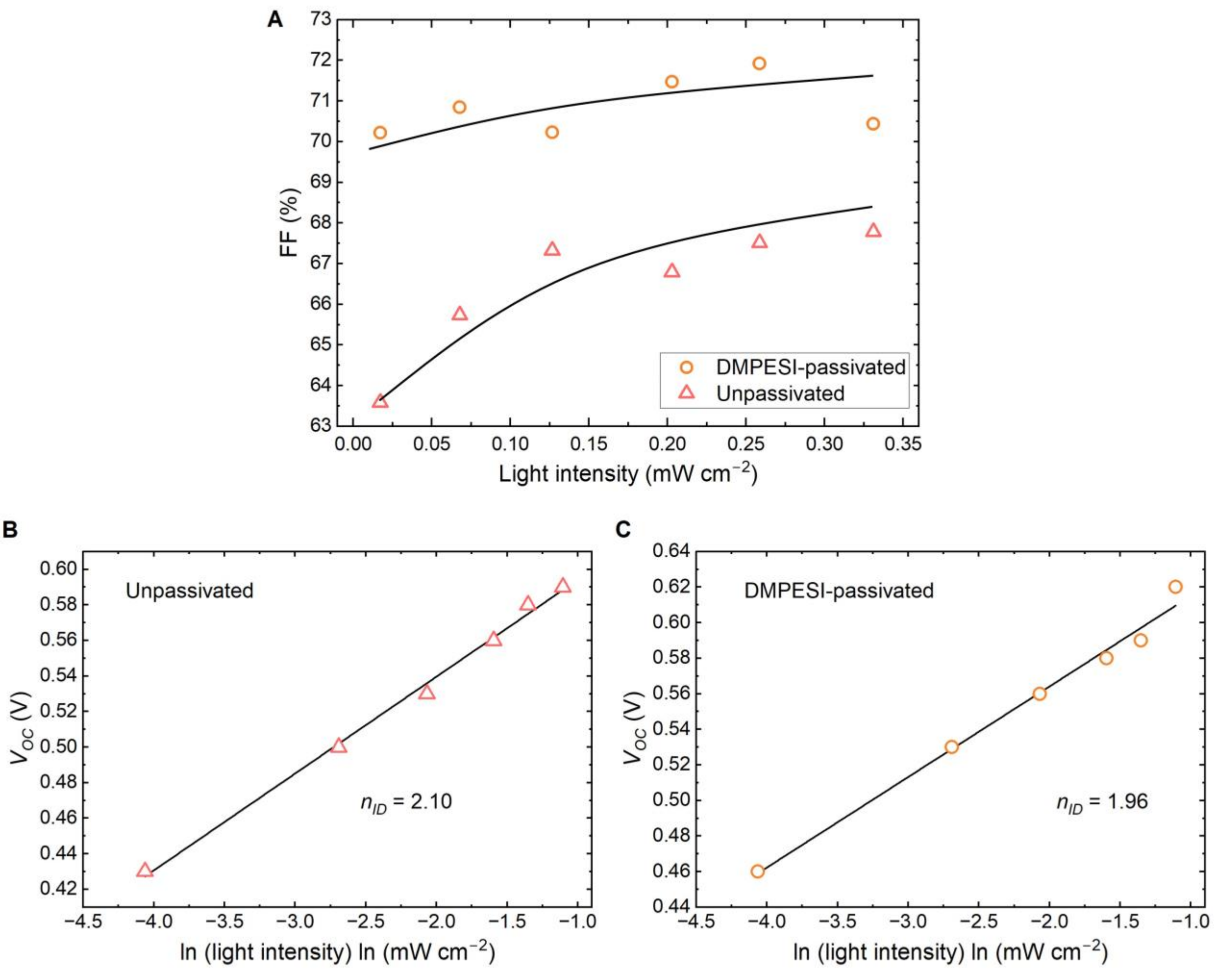

Figure S16. Indoor light-intensity-dependent photovoltaic characteristics (50–1000 lux) of unpassivated and DMPESI-passivated CsMAFA-Sb:Bi devices. (A) Fill factor (FF) trends as a function of light intensity. Semi-logarithmic plots of V_{OC} vs. light intensity for (B) unpassivated and (C) DMPESI-passivated CsMAFA-Sb:Bi devices. The lowered FF losses and reduced ideality factor observed in the passivated devices indicate suppressed defect-related recombination, attributed to effective passivation of the CsMAFA-Sb:Bi layer.

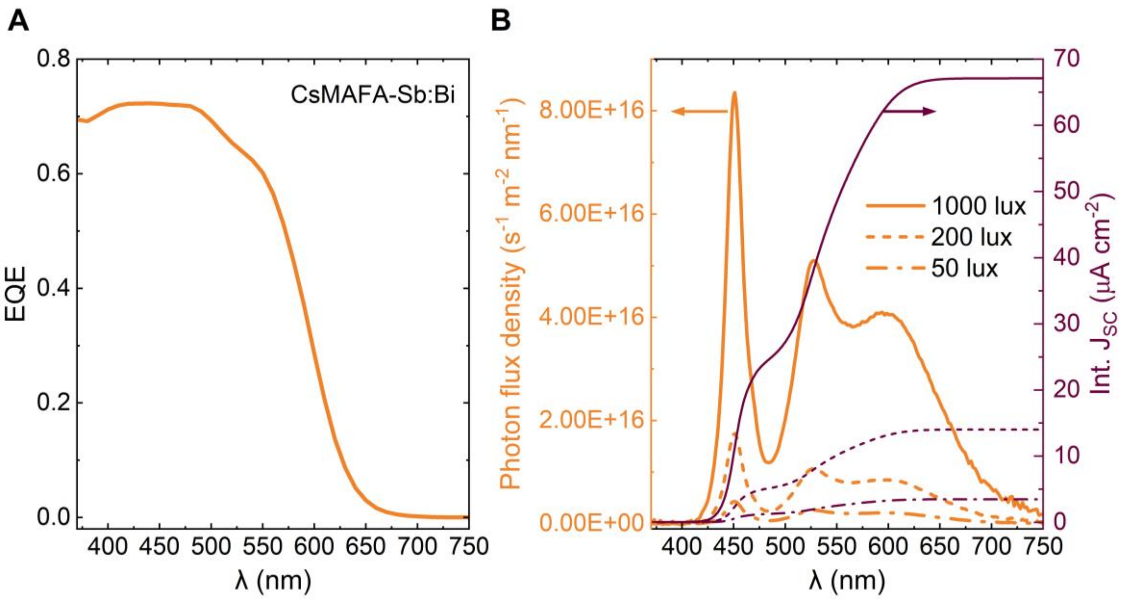

Figure S17. (A) External quantum efficiency (EQE) spectrum of the CsMAFA-Sb:Bi champion device with the architecture FTO/c-TiO_2/m-TiO_2+CsMAFA-Sb:Bi/DMPESI/Spiro-OMeTAD/Au. (B) Photon flux density spectra of the 6500 K white LED (WLED) at illumination levels of 1000, 200, and 50 lux, along with the corresponding integrated short-circuit current density (J_{SC}) profiles.

A mismatch of less than 10% was observed between the J_{SC} values obtained from current density-voltage (*J–V*) measurements and external quantum efficiency (EQE) integration under WLED illumination (Table S4), confirming good measurement consistency.

***Note:** EQE measurements were performed under standard dark conditions using a monochromatic light source and numerically integrated with the spectral photon flux density profiles of the WLED at 1000, 200, and 50 lux to estimate indoor J_{SC} values. This is a widely accepted approach in indoor PV characterization, particularly when white-light biasing is unavailable. The resulting J_{SC} values show good agreement with those derived from J–V measurements (**Table S4**). EQE measurements under light bias at varying illumination levels were not conducted due to current instrumental constraints. We aim to implement EQE characterization under light bias in future studies to more comprehensively assess device performance under realistic indoor conditions.*

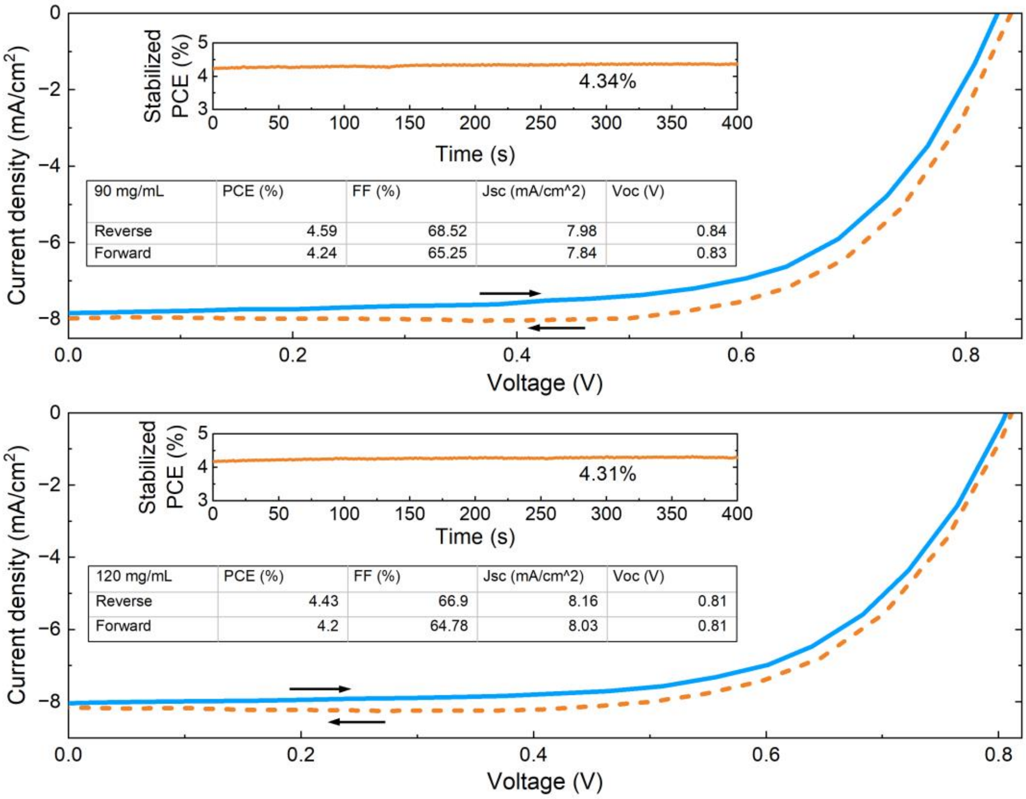

Figure S18. Current density–voltage (*J–V*) curves of the champion devices with the architecture FTO/c-TiO_2/m-TiO_2+CsMAFA-Sb:Bi/DMPESI/Spiro-OMeTAD/Au, fabricated using m-TiO_2 precursor solutions at concentrations of 90 mg/mL (top panel) and 120 mg/mL (bottom panel), measured under 1-Sun illumination. Arrows indicate the *J–V* scan direction. Insets show the corresponding device parameters and stabilized PCE curves, obtained via maximum power point tracking. The J_{SC} values derived from integrating the EQE spectra over the 1-sun spectrum were 8.4 mA/cm^2 (J_{SC} mismatch: 5.3%) for the 90 mg/mL device, and 8.5 mA/cm^2 (J_{SC} mismatch: 4.2%) for the 120 mg/mL device.

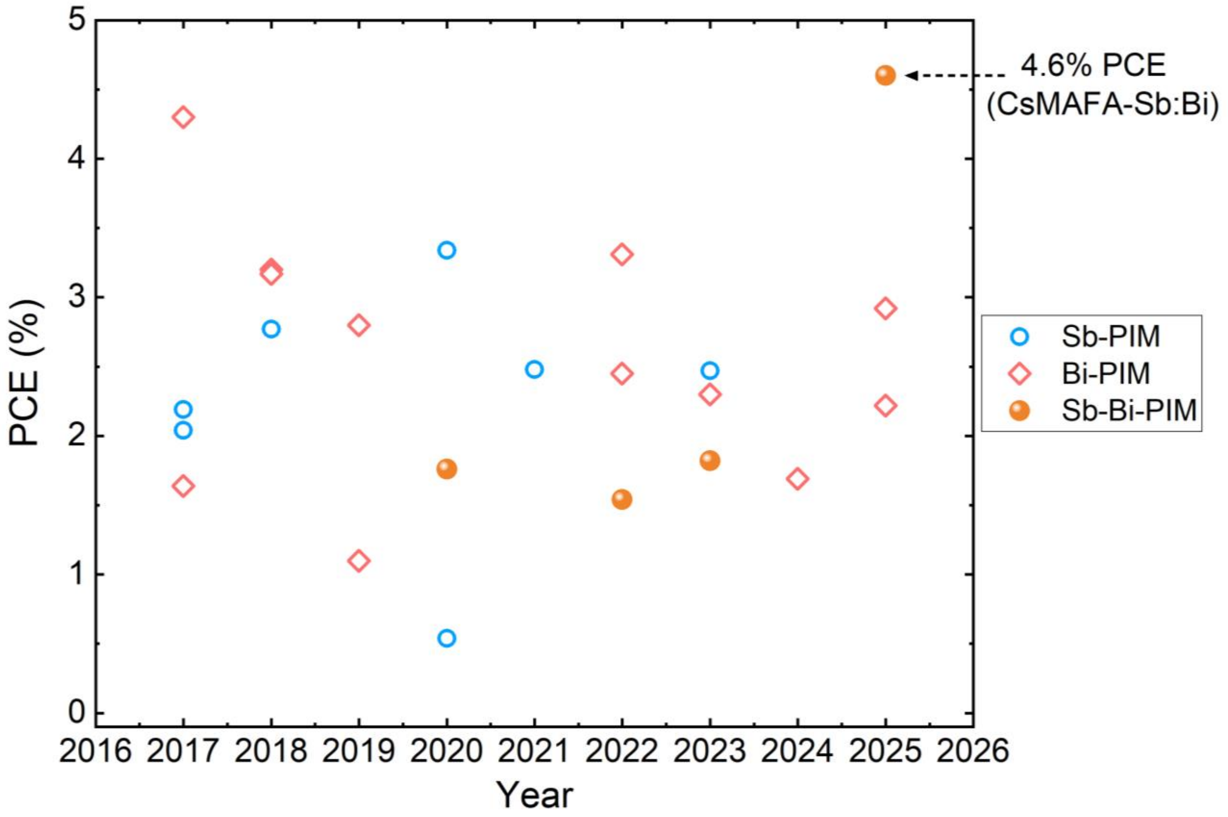

Figure S19. Evolution of power conversion efficiency (PCE) over time for wide-bandgap (>1.7 eV) Sb-, Bi-, and Sb–Bi-based halide perovskite-inspired materials (PIMs) under 1-Sun illumination, plotted by year of publication. The comparison includes only devices where the PIM serves as the sole light absorber and highlights the performance of our CsMAFA-Sb:Bi device relative to previously reported values. Data compiled from References.[7–13]

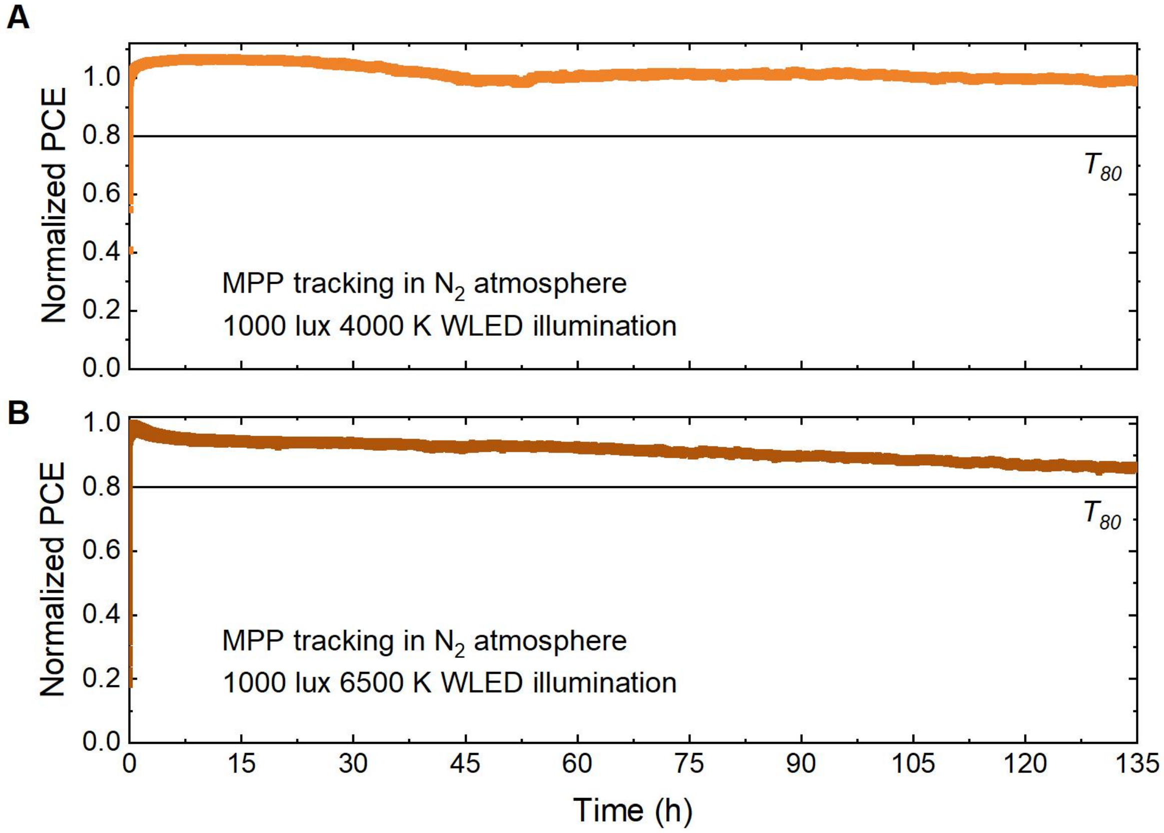

Figure S20. Normalized power conversion efficiency (PCE) of a CsMAFA-Sb:Bi device measured under continuous 1000 lux WLED illumination of color temperatures of 4000 K (A) and 6500 K (B), in a nitrogen atmosphere, using maximum power point (MPP) tracking. T_{80} refers to the time required for the device's PCE to drop to 80% of its initial value (serves as a standard metric for operational stability).

Note: *MPP tracking was performed on CsMAFA-Sb:Bi devices under 1000 lux WLED illumination to assess early-stage operational stability. Under 4000 K WLED, the device retained nearly 100% of its initial performance over 135 hours (**Figure S20A**). A second device tested under 6500 K WLED retained over 85% of its initial PCE over the same duration (**Figure S20B**). This difference in degradation behavior highlights the potential influence of WLED spectral composition on long-term device performance. A more systematic investigation is planned for future work to better understand these effects.*

Supplementary Note 4: Power Output Relevance to IoT Applications

The demonstrated power output densities of our CsMAFA-Sb:Bi devices under indoor lighting conditions are:

- **1000 lux:** 31.6 μW cm^{-2}
- **200 lux:** 5.5 μW cm^{-2}
- **50 lux:** 1.1 μW cm^{-2}

These values align well with the operational demands of many low-power IoT applications. For example:

- **Bluetooth Low Energy (BLE) sensors** typically operate in the range of 10–50 μW during idle-to-transmit transitions.
- **Electronic shelf labels (ESLs)** in retail settings draw 5–20 μW on average.
- **Environmental monitors** (e.g., temperature, humidity, CO_2) can function with 10–100 μW, depending on sampling rate and communication protocol.
- **RFID sensors and e-ink displays** often require even lower power, particularly when duty-cycled or event-triggered.

With a current active device area of 0.1 cm^2, the absolute power outputs are 3.16 μW (1000 lux), 0.55 μW (200 lux), and 0.11 μW (50 lux). However, since output increases with the device area, even modestly sized modules (1–10 cm^2) could yield tens to hundreds of μW, making them well-suited for powering intermittently active or always-on IoT nodes under ambient indoor lighting.

This supports the real-world applicability of our >10% indoor PCE achievement—not only as a milestone in lead-free IPV performance but also as a meaningful step toward integrated, battery-light or battery-free energy solutions for IoT electronics.

Supplementary Note 5: Practical Integration Considerations for IoT Applications

While this study focuses on demonstrating high-efficiency IPV performance of the low-toxicity PIMs, practical deployment in IoT systems involves several considerations beyond raw device metrics. These include:

Voltage Matching: Typical IoT nodes operate at 1.8–3.3 V, requiring either series connection of multiple sub-cm^2 cells or use of DC–DC boost converters. With a device V_{OC} of ~0.5–0.65 V (depending on illumination), achieving system-level voltage presently demands several cells in series. While this is standard in indoor energy harvesting, it adds design complexity and potential interconnect losses. This constraint can be resolved in future device iterations through interface engineering and compositional tuning to raise V_{OC}.

Power Management Circuits: Energy harvesting ICs (e.g., BQ25570, AEM10941) enable maximum power point tracking (MPPT), cold start, and load regulation under low-light conditions. The demonstrated early-stage operational stability of our devices—maintaining performance over 135 hours under 1000 lux illumination—supports their compatibility with such indoor power management solutions.

Energy Storage Integration: Storage components like microcapacitors or thin-film batteries help buffer brief periods of light interruption. The power generated by our devices (Table 1) is sufficient to continuously charge small energy storage components under typical indoor lighting conditions.

Lighting Variability: Indoor light is inherently variable. However, many low-power IoT devices operate in duty-cycled or event-driven modes, making them resilient to such fluctuations. The ability of our devices to deliver stable output under varying lux levels supports their applicability in these scenarios.

Taken together, these considerations strengthen the practical relevance of our Sb/Bi PIM-based IPVs. While this work emphasizes IPV device-level advancements, integrating them into fully functional, self-powered IoT systems represents a key direction for future research.

Supporting Tables

Table S1. Average photovoltaic parameters (from 20 devices per group) for unpassivated and DMPESI-passivated CsMAFA-Sb:Bi devices with the architecture FTO/c-TiO_2/m-TiO_2+CsMAFA-Sb:Bi/DMPESI/Spiro-OMeTAD/Au under 1000 lux WLED illumination (6500 K). The highest value for each parameter is shown in parentheses.

Device parameter	Unpassivated	DMPESI-passivated
PCE (%)	8.85+/-0.30 (9.40)	9.7+/-0.29 (10.11)
J_{SC} (μA cm^{-2})	73.7+/-2.64 (77.4)	75.9+/-2.30 (79.7)
V_{OC} (V)	0.622+/-0.01 (0.639)	0.636+/-0.01 (0.650)
FF (%)	62.9+/-2.56 (66.4)	66.5+/-2.43 (71.2)

Table S2. Binding energy values of various elements in unpassivated and DMPESI-passivated CsMAFA-Sb:Bi films, along with the corresponding shifts (ΔE) in binding energy.

XPS peak	Unpassivated CsMAFA-Sb:Bi	DMPESI-passivated CsMAFA-Sb:Bi	Shift (ΔE) in peak position (eV)
$I3d_{5/2}$	619.41 eV	619.29 eV	0.12
$I3d_{3/2}$	630.91 eV	630.78 eV	0.13
$Sb3d_{5/2}$	530.25 eV	530.13 eV	0.12
$Sb3d_{3/2}$	539.61 eV	539.50 eV	0.11
$Bi4f_{7/2}$	158.98 eV	158.92 eV	0.06
$Bi4f_{5/2}$	164.31 eV	164.18 eV	0.13
$Cs3d_{5/2}$	725.05 eV	724.92 eV	0.14
$Cs3d_{3/2}$	738.91 eV	738.88 eV	0.03
Cl $2p_{3/2}$	198.98 eV	198.66 eV	0.32
Cl $2p_{1/2}$	201.22 eV	201.21 eV	0.02
N 1s	400.39 eV	400.27 eV	0.12
N 1s	402.40 eV	402.10 eV	0.30

Table S3. Grain size analysis and roughness parameters for unpassivated and DMPESI-passivated CsMAFA-Sb:Bi film samples based on AFM measurements. Here the roughness was measured from a 128 x 512 section of the sample in the centre of the frame. RMS = root mean square. The mean size is calculated by averaging the equivalent square sides. Its square is not, in general, equal to the mean area.

Sample	Mean Grain Size (nm)	RMS Roughness (nm)	RMS Waviness (nm)
CsMAFA-Sb:Bi (on meso TiO_2), on glass	220	10.08	23.82
DMPESI-passivated CsMAFA-Sb:Bi (on meso TiO_2), on glass	193	3.47	10.87

"Surface texture typically consists of waviness and roughness. Waviness refers to longer wavelengths on the surface which is related to surface preparation and finishing methods. Roughness refers to fluctuations on shorter wavelengths or irregularities of the surface." From: https://www.parksystems.com/en/learning-center/lc-detail.learning843

Table S4. Comparison of short-circuit current densities (J_{SC}) derived from reverse and forward J–V scans and EQE integration under 6500 K white LED (WLED) illumination at 1000, 200, and 50 lux. J_{SC} mismatch percentages are calculated relative to EQE-derived J_{SC} values, and the average mismatch provides an overall measure of consistency across methods.

6500 K WLED	J_{SC} (REV J-V) (μA/cm^2)	J_{SC} (FOR J-V) (μA/cm^2)	J_{SC} from EQE (μA/cm^2)	J_{SC} mismatch (REV J-V) %	J_{SC} mismatch (FOR J-V) %	Average J_{SC} mismatch %
1000 lux	73.8	71.4	67.1	9.9	6.4	8.1
200 lux	14.3	13.9	14.0	1.9	0.9	1.4
50 lux	3.6	3.2	3.5	3.4	8.0	5.7

Table S5. The photovoltaic performance metrics of the champion CsMAFA-Sb:Bi device measured under 1000 lux WLED illumination across different color temperatures.

WLED color temperature	PCE (%)
6500 K	10.11
5000 K	9.1
4000 K	8.4
2700 K	7.2

REFERENCES

(1) Lamminen, N.; Karlsson, J.; Kumar, R.; Viswanath, N. S. M.; Lal, S.; Fasulo, F.; Righetto, M.; Krishnaiah, M.; Lahtonen, K.; Tewari, A.; Katerski, A.; Lahtinen, J.; Acik, I. O.; Johansson, E. M. J.; Muñoz-García, A. B.; Pavone, M.; Herz, L. M.; Grandhi, G. K.; Vivo, P. The Promise of Operational Stability in Pnictogen-Based Perovskite-Inspired Solar Cells. *EES Sol.* **2025**, *1* (2), 139–156.

(2) Nečas, D. and Klapetek, P., 2012. Gwyddion: an open-source software for SPM data analysis. *Open Physics*, **2012**, *10(1)*, 181-188.

(3) Suo, J.; Yang, B.; Mosconi, E.; Bogachuk, D.; Doherty, T. A. S.; Frohna, K.; Kubicki, D. J.; Fu, F.; Kim, Y.; Er-Raji, O.; Zhang, T.; Baldinelli, L.; Wagner, L.; Tiwari, A. N.; Gao, F.; Hinsch, A.; Stranks, S. D.; De Angelis, F.; Hagfeldt, A. Multifunctional Sulfonium-Based Treatment for Perovskite Solar Cells with Less than 1% Efficiency Loss over 4,500-h Operational Stability Tests. *Nat. Energy* **2024**, *9* (2), 172–183.

(4) Xu, T.; Liu, S.; Seok, S. I.; Xiang, W. Surface Chemistry-Induced Reconstruction of Inorganic Perovskites for Efficient and Stable Inverted Solar Cells. *Joule* **2025**, *9* (4), 101826.

(5) Suo, J.; Yang, B.; Mosconi, E.; Choi, H.-S.; Kim, Y.; Zakeeruddin, S. M.; De Angelis, F.; Grätzel, M.; Kim, H.-S.; Hagfeldt, A. Surface Reconstruction Engineering with Synergistic Effect of Mixed-Salt Passivation Treatment toward Efficient and Stable Perovskite Solar Cells. *Adv. Funct. Mater.* **2021**, *31* (34), 2102902.

(6) Miller, E. M.; Zhao, Y.; Mercado, C. C.; Saha, S. K.; Luther, J. M.; Zhu, K.; Stevanović, V.; Perkins, C. L.; Lagemaat, J. van de. Substrate-Controlled Band Positions in $CH_3NH_3PbI_3$ Perovskite Films. *Phys. Chem. Chem. Phys.* **2014**, *16* (40), 22122–22130.

(7) Grandhi, G.K.; Hardy, D.; Krishnaiah, M.; Vargas, B.; Al-Anesi, B.; Suryawanshi, M.P.; Solis-Ibarra, D.; Gao, F.; Hoye, R.L.; Vivo, P. Wide-bandgap perovskite-inspired materials: defect-driven challenges for high-performance optoelectronics. *Adv. Funct. Mater.* **2024**, *34*(50), 2307441.

(8) Singh, M.; Akash; Tiwari, J.P. Solar Cells Based on Pb-Free and Perovskite-Inspired Materials: Futuristic of Perovskite Solar Cells. *ACS Appl. Energy Mater*. **2024**, *7*(22), 10212-10229.

(9) Boopathi, K.M.; Karuppuswamy, P.; Singh, A.; Hanmandlu, C.; Lin, L.; Abbas, S.A.; Chang, C.C.; Wang, P.C.; Li, G.; Chu, C.W. Solution-processable antimony-based light-absorbing materials beyond lead halide perovskites. *J. Mater. Chem. A*. **2017**, *5*(39), 20843-20850.

(10) Raval, N.A.; Kheraj, V. Evolution and state-of-the-art development of antimony-based perovskites material-system for solar photovoltaics: A comprehensive review. *Solar Energy*. **2025**, *286*, 113128.

(11) Zhang, Z.; Li, X.; Xia, X.; Wang, Z.; Huang, Z.; Lei, B.; Gao, Y. High-quality $(CH_3NH_3)_3Bi_2I_9$ film-based solar cells: pushing efficiency up to 1.64%. *J. Phys. Chem. Lett.* **2017**, *8*(17), 4300-4307.

(12) Magdalin, A.E.; Nixon, P.D.; Jayaseelan, E.; Sivakumar, M.; Devi, S.K.N.; Subathra, M.S.P.; Kumar, N.M.; Ananthi, N. Development of lead-free perovskite solar cells: Opportunities, challenges, and future technologies. *Results in Engineering*. **2023**, *20*, 101438.

(13) Li, B.; Wu, X.; Zhang, S.; Li, Z.; Gao, D.; Chen, X.; Xiao, S.; Chueh, C.C.; Jen, A.K.Y.; Zhu, Z. Efficient and stable $Cs_2AgBiBr_6$ double perovskite solar cells through in-situ surface modulation. *Chemical Engineering Journal*. **2022**, *446*, 137144.
